# Automated tumour budding quantification by machine learning augments TNM staging in muscle-invasive bladder cancer prognosis

**DOI:** 10.1038/s41598-019-41595-2

**Published:** 2019-03-26

**Authors:** Nicolas Brieu, Christos G. Gavriel, Ines P. Nearchou, David J. Harrison, Günter Schmidt, Peter D. Caie

**Affiliations:** 1 Definiens AG, Bernhard-Wicki-Straße 5, 80636 München, Germany; 2 School of Medicine, University of St Andrews, North Haugh, St Andrews, Fife KY16 9TF UK

## Abstract

Tumour budding has been described as an independent prognostic feature in several tumour types. We report for the first time the relationship between tumour budding and survival evaluated in patients with muscle invasive bladder cancer. A machine learning-based methodology was applied to accurately quantify tumour buds across immunofluorescence labelled whole slide images from 100 muscle invasive bladder cancer patients. Furthermore, tumour budding was found to be correlated to TNM (p = 0.00089) and pT (p = 0.0078) staging. A novel classification and regression tree model was constructed to stratify all stage II, III, and IV patients into three new staging criteria based on disease specific survival. For the stratification of non-metastatic patients into high or low risk of disease specific death, our decision tree model reported that tumour budding was the most significant feature (HR = 2.59, p = 0.0091), and no clinical feature was utilised to categorise these patients. Our findings demonstrate that tumour budding, quantified using automated image analysis provides prognostic value for muscle invasive bladder cancer patients and a better model fit than TNM staging.

## Introduction

Muscle-invasive bladder cancers (MIBC) are classified as having grown into or through the muscle layers of the bladder wall^1^ however are phenotypically heterogeneous. Current clinical guidelines for both prognosis and treatment strategies are based on Tumour Node Metastasis (TNM) staging system; of which MIBC cancers are comprised of pT2–pT4 tumours, with or without nodal and distant metastasis^2^. Patients with MIBC are recommended for radical cystectomy with pelvic lymph node dissection. Approximately 25% of newly diagnosed bladder cancer patients present with muscle invasive disease. In contrast to non-muscle invasive bladder cancer (NMIBC), MIBC is highly aggressive with a high incidence of metastasis and a poor prognosis where 5-year survival rates vary between 30 and 50%^3,4^.

Despite recent research into novel treatment and surgical strategies the mortality rates and prognosis of MIBC patients have remained immutable over the past 30 years^3,5^. In addition to inter- and intra-reporter variability, TNM staging may not adequately encompass the complex and dynamic behaviour of the disease^6–10^. There is therefore a need to substantially improve patient stratification and earlier definitive treatment for high risk MIBC patients. A morphological analysis of the different patterns of tumour invasion may hold important pathological information, which could allow for a better risk stratification of patients with MIBC.

Tumour budding (TB) is defined as discrete clusters of up to four cancer cells that reflects an infiltrative invasive growth pattern^11,12^. Most studies have shown that a higher density of TB is typically present at the invasive front (peritumoural budding) compared to tumour core (intratumoural budding)^12–17^. It has been suggested that TB reflects an early step in progression towards metastasis due to their cell’s reported phenotypic epithelial to mesenchymal transition (EMT)^18–20^. Despite reports of observer variability, studies in other cancer types have continuously demonstrated the significant association of TB with adverse clinicopathological features and the independent significance of TB as a prognostic factor^21–25^. Information about the role of TB in bladder cancer is scarce^26^ and it has not yet been reported to be associated with disease specific survival (DSS) in MIBC.

In this study, we evaluate whether TB can more precisely predict MIBC patients at a low or medium risk of disease specific death than current clinical guidelines based on TNM staging. To this end, we introduce an image analysis solution for the detection of tumour buds, which following from our previous works^27,28^, is based on the automated detection of both the nuclei and the segmentation and classification of cancer cell clusters. Our automated solution enables the quantification of TB at the patient level via the computation of seven image analysis features, while eliminating inter-observer variability. Survival analysis provided evidence of the prognostic value of TB quantification in MIBC patients and showed that a combination of clinicopathological features with TB predicted patient survival more precisely than the standard TNM staging.

## Results

### Patient clinicopathological data

This study comprised 100 MIBC patients with a median age at the time of surgical resection of 68 years (range; 29–87 years). The follow-up time was up to 113.13 months. DSS was gathered from NHS Scotland’s patient database and was defined by the patient’s cause of death being directly from their bladder cancer. Median DSS is 23.63 months. In terms of TNM staging, 25% of patients were diagnosed with stage II, 41% with stage IIIA, 4% with stage IIIB and 30% with stage IV. No positive lymph nodes were found in 78 patients and 1–2 lymph nodes contained cancer in 22 patients. Full patient characteristics can be found in Table 1.

**Table 1:** Clinicopathological features evaluated in this study.

Features	Patients number N = 100 (%)					
Age	< 70 (55%)				≥ 70 (45%)	
Gender	Male (59%)				Female (41%)	
TNM staging	II (25%)		IIIA (41%)		IIIB (4%)	IV (30%)
pT stage	2a (9%)	2b (18%)	3a (19%)	3b (30%)	4a (13%)	4b (11%)
Metastasis	Absent (71%)			Present (29%)		
Lymph node status	N0 (78%)		N1 (14%)		N2 (8%)	
Patient outcome	Survived (46%)		Died of disease (54%)			
Treatment (cf. Table S4)	Tr1 (11%)	Tr2 (9%)	Tr3 (13%)	Tr4 (9%)	Tr5 (50%)	Other (12%)
Grade	G2 (11%)		G2-3 (2%)		G3 (87%)	
Growth pattern	Solid (70%)		Solid/Papillary (16%)		Papillary (12%)	Other (2%)

The explanation of treatment option codes are provided in Supplementary Table S4.

### Survival statistics on the MIBC cohort

TNM staging, returned the lowest log rank p-value (p = 4.76E^−11^) and highest hazard ratio (HR = 5.44, p = 2.53E^−09^) than any other image analysis or clinical feature. Of all the treatment combinations, as displayed in Supplementary Table S4, treatment with only Mytomycin C was the sole treatment significantly associated with survival (HR = 0.21, p = 0.018) while neither the growth pattern nor the grade were prognostic for survival in our cohort. The total number of tumour buds in the detected tumour core (‘number of TB in core’) reported the highest significance for MIBC patient survival by both the log rank test (p = 9.97E^−06^) and Cox-regression (HR = 3.22, p = 2.67E^−05^) than any other TB quantification method. Full survival univariate statistics for each analysed parameter are stated in Table 2.

**Table 2:** Results of univariate log rank test and univariate Cox regression analysis of clinical and image analysis features associated with disease specific survival in all MIBC patients (N = 100).

Features	Log rank test	Cox regression	
	p value/q value	Hazard Ratio [95% CI]	p value/q value
TNM stage	**4**.**76E**^−11^***	**5**.**44 [3**.**11 9**.**49]**	**2**.**53E**^−09^***
pT stage	**6**.**18E**^−06^***	**3**.**59 [1**.**99 6**.**47]**	**2**.**15E**^−05^***
Lymph node status	0.0169*	1.99 [1.11 3.54]	0.0191*
Metastasis	**4**.**76E**^−11^***	**5**.**44 [3**.**11 9**.**49]**	**2**.**53E**^−09^***
Grade	0.5724	1.27 [0.54 2.98]	0.5780
Growth Pattern	0.3288	0.63 [0.25 1.59]	0.3326
Treatment	0.0178*	0.21 [0.05 0.87]	0.0318 *
Gender	0.6555	0.88 [0.51 1.51]	0.6545
Age	0.1932	0.67 [0.37 1.22]	0.1974
Number of TB in core	**9**.**97E**^−06^***/**6**.**70 E**^**−05**^	3.22 [1.87 5.57]	**2**.**67E**^−05^***/**1**.**87E**^**−04**^
Number of TB in invasive front	0.12687/0.1501	1.51 [0.88 2.60]	0.1300/0.1517
Density of TB in core	0.01312*/0.0306	1.99 [1.14 3.46]	0.0150*/0.0350
Density of TB in invasive front	0.05876/0.0822	1.71 [0.97 3.03]	0.0619/0.0867
Number of TB in a single 0.785mm2 field of view	0.24322/0.24322	1.41 [0.78 2.53]	0.2458/0.2458
Number of TB in ten 0.785mm2 fields of view	0.0077**/0.0269	2.04 [1.19 3.49]	0.0091**/0.0319
Number of TB in ten 0.238mm2 fields of view	0.03919*/0.0685	1.85 [1.02 3.37]	0.04214*/0.0737

Reported p-values and hazard ratios are obtained for each feature through leave-one-out pre-validation of the optimal separation cut-off used to separate the patients into two optimal low/high sub-groups. (***) indicates p < 0.001, (**) p < 0.01 and (*) p < 0.05. For the seven tumour budding features, p values corrected for the multiple tumour budding hypothesis using the most conservative Benjamini method (q-values) are reported together with the original p values.

### Association of tumour budding with clinicopathological data

The chi-squared (χ^2^) test of independence was used to examine associations between clinicopathological data and the ‘number of TB in the core’ (see Supplementary Table S2). High TB was found to be correlated with higher TNM stage (χ^2^ = 11.037, p = 0.00089), and pT stage (χ^2^ = 7.065, p = 0.0078) which were also the two most significant clinicopathological features associated with patient survival and were previously demonstrated predictors of patient outcome. No other clinicopathological feature was associated with TB.

### Survival Decision Tree

**Figure 1:**
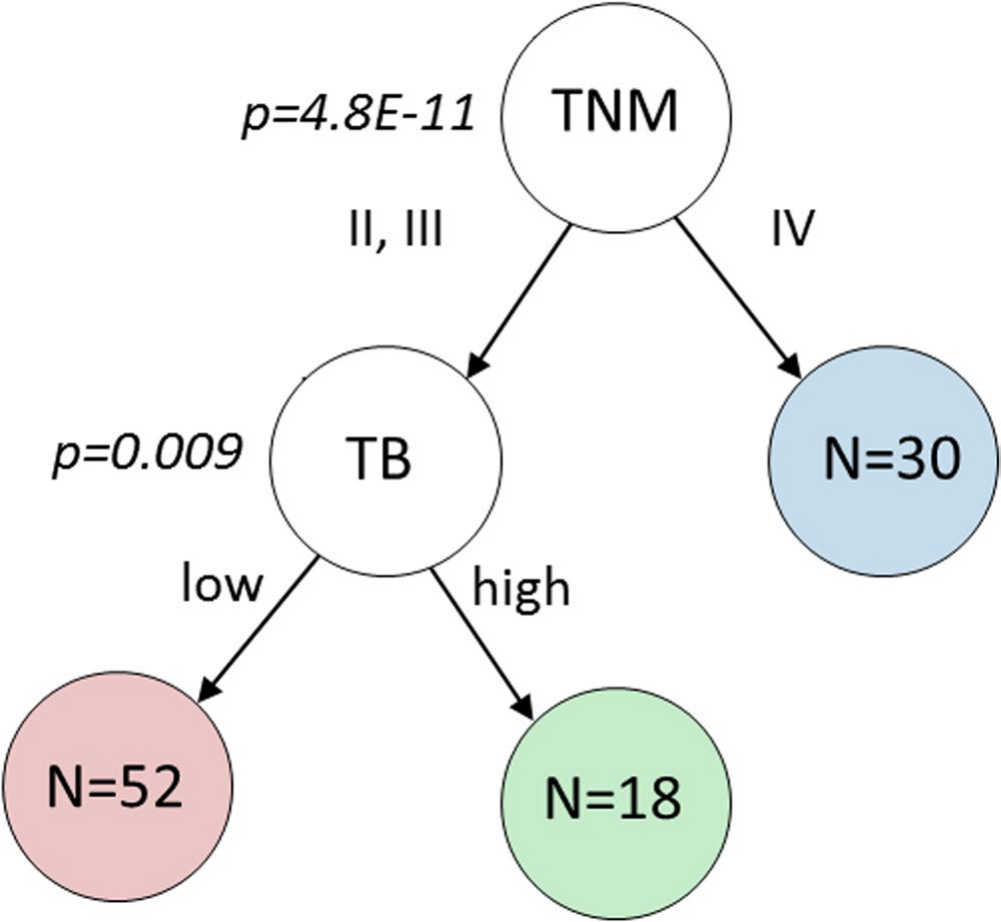
Proposed survival decision tree grouping of MIBC patients into three distinct groups, which results in the proposed ‘TB stage model’. The leave one out cross-validated log rank test p value between the two resulting branches is indicated at each node. The first decision is based on the clinical parameter TNM stage and the second decision on the feature ‘number of TB in core’. The number of patients is indicated for each resulting group/leaf.

In order to assess if a combination of clinicopathological and TB features could predict patient survival more precisely than the standard TNM staging, we performed a survival decision tree analysis (see Fig. 1 and Methods for more details). As listed in Table 2, all seven tumour bud features were entered as input into the survival decision tree alongside all clinicopathological parameters. The first reported split of the decision tree separated patients with (stage II and III) from those with metastasis (stage IV) by the use of the TNM staging parameter (HR = 5.44, p = 2.53E^−09^). The resulting sub-group of stage II and III MIBC patients without metastasis (N = 70) was further optimally separated by the ‘number of TB in core’ feature. The associated log rank p value (p = 0.0091) and hazard ratio (HR = 2.59, p = 0.0119) showed the statistical significance of this second split against other TB features and clinicopathological parameters (see Supplementary Table S1). To prevent overfitting, leave one-out cross-validation was applied. Through the N = 100 leave-one-out pre-validation runs, TNM was systematically chosen to take the first decision on all patients. On the non-metastatic patients, the feature ‘number of TB in core’ was chosen in 97 of the 100 runs whereas the parameter pT stage was only chosen in the three remaining runs. To ensure the unicity of the model, we fixed the second decision feature for the branch analysing non-metastatic patients to ‘number of TB in core’ and cross-validated the associated cut-off value. The resulting survival decision model is denoted as ‘TB stage model’ in the remaining of this work. As detailed in the Methods, the TB stage model splits the MIBC patients into three groups that we arbitrarily denote as II’, III’ and IV. More precisely, and as shown by the association table between the TNM and TB stage models (see Supplementary Table S5), the proposed TB stage model reassigns a subset of TNM stage III patients (N = 27) into stage II’.

### Novel TB stage model

**Figure 2:**
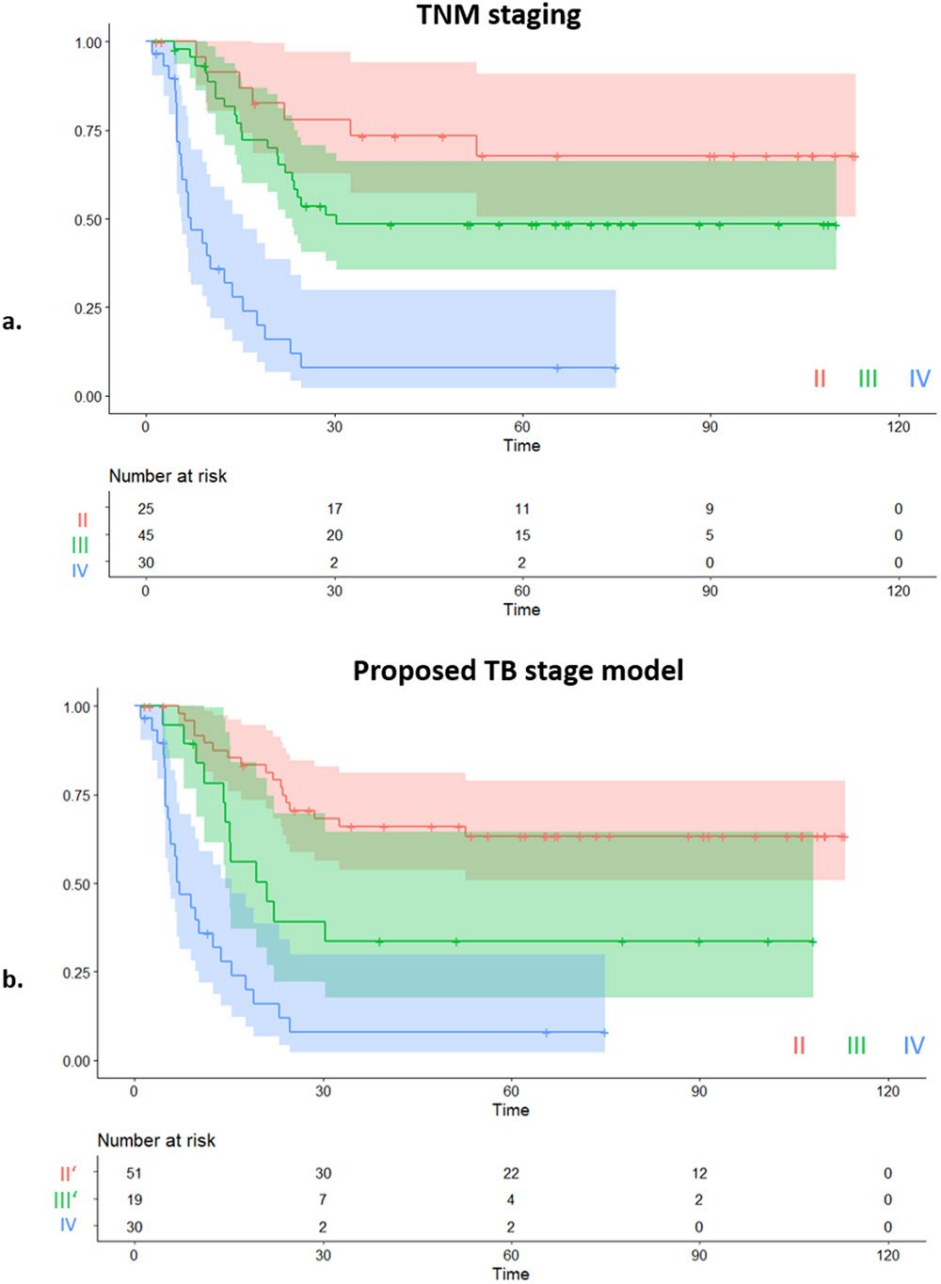
(**a**) Kaplan-Meier plot and risk table of disease specific survival (DSS) for MIBC patients in dependence of TNM staging (II, III, IV). (**b**) Kaplan-Meier plot and risk table of DSS for MIBC patients utilising the TNM stage model and the novel ‘TB stage model’.

Results of categorical Cox-regression analysis on the TB stage model and on the clinical TNM staging are reported in Table 3. Groups II’ (TB stage model) and II (clinical TNM model) are taken as baseline respectively. Corresponding Kaplan Meier curves are displayed in Fig. 2. We observed that the proposed novel model yields statistically significant separation (p < 0.05) for both stage III’ and stage IV patients. This indicates a strong relationship between the model’s proposed staging and an increased risk of disease specific death. The TB model stage III’ returned a HR of 2.48 with stage II’ as baseline, as compared with the clinical TNM stage III returning a HR of 1.92 with clinical stage II as baseline. Furthermore, the confidence interval for HR on clinical TNM stage III included 1 and the separation between TNM stage II and stage III patients is not significant (p = 0.13). This observation is confirmed by the outputs of the three overall tests (Likelihood ratio, Wald and log rank). While the p values for both the clinical TNM and the proposed TB model’s staging, for the aforementioned statistical tests, are highly significant (p < 1E-07), the proposed staging yields larger test statistics. This indicates a stronger rejection of the null hypothesis that the stage has no effect on the survival and it suggests a better model fit to survival than the standard TNM stage.

**Table 3:** Results of Cox regression analysis on all MIBC patients stratified into three groups (II, III and IV) using the standard TNM staging and the proposed model respectively, with their respective stage II as reference.

Model	Cox regression			Likelihood ratio test	Wald test	Log rank test
	Factor	HR [95% CI]	p value/q value			
Clinical TNM staging	Stage III	1.92 [0.82 4.51]	0.13	G = 34.1 p = 3.94E^−08^	z^2 = ^36.2 p = 1.41E^−08^	lr = 44.95 p = 1.74E^−10^
	Stage IV	8.66 [3.65 20.28]	8.4E^−07^**			
TB stage model	**Stage III**	**2.48 [1.18 5.21]**	0.017*/0.119	G = 36.9 p = 9.78E^−09^ q = 6.85E^−08^	z^2 = ^37.83 p = 6.09E^−09^ q = 4.26E^−08^	lr = 47.35 p = 5.22E^−11^ q = 3.65E^−10^
	Stage IV	7.33 [3.87 13.89]	1.0E^−09^**/7.0E^−09^			

Two degrees of freedom are considered for overall tests. P values of the TB stage model are corrected (q values) for multiple hypothesis testing corresponding to the (m = 7) features used for TB quantification.

### Multiple hypothesis testing

The main test hypothesis in this study is that TB predicts disease specific survival in MIBC patients. Characterising TB with multiple approaches leads us to define in practice m = 7 test hypotheses, i.e. one hypothesis for each of the seven analysed TB features (as detailed in Table 2) e.g. H1: “Number of TB in core” predicts survival, H2: “Number of TB in invasive front” predicts survival, H3: “Density of TB in Core” predicts survival. Correcting the p values reported in the previous paragraphs using the most conservative Benjamini method yields the ‘number of TB in Core’ to keep significance below α = 0.001 on all MIBC patients (see Table 2, q-values). When applying p value correction to non-metastatic patients (see Supplementary Table S1), the corrected log rank p value of ‘number of TB in Core’ is very close to the expected level of significance (p = 0.064 vs. p = 0.05). While more realistic p values could be computed after the estimation of the expected proportion of true null p-values π_0_, we found the number of p values to be too small in practice to enable such estimation. With the Benjamini methodology being the most conservative, we can however conclude that the reported corrected p values are upper bounds of the actual ones.

## Discussion

To investigate whether the presence of TB predicts survival in patients with MIBC, we employed immunofluorescence, image analysis and machine learning for the quantification and statistical analysis of TB, in an accurate, fully automated and standardized manner. We found that the incorporation of TB features improved the accuracy of the prediction of DSS compared to standard TNM staging alone. TB has been identified as a predictor of poor outcome in many types of cancer such as pancreatic, breast, colorectal and lung cancer^21–23,29^. As far as we are aware, we present the first evidence of TB holding prognostic significance in MIBC.

### Methodologies for TB quantification

Despite TB being reported as an independent predictor of patient outcome in several disease areas, it has not been accepted as a core item in the clinical guidelines^30,31^ and consequently not regularly used in patient management. One of the reasons for this is the variety of TB quantification methods employed in individual studies; however, this standardisation issue is being addressed for colon cancer by the ITBCC^12^. In order to identify the optimal methodology to quantify TB in MIBC we reported on the number and density (per area pixel) of tumours buds across four different methods. Methods 1 and 2 were based on work by Karamitopoulou E *et al*.^21^ and Ueno H *et al*.^32^ and the recommendations of ITBCC^12^. Method 3 was inspired by Lohneis P *et al*.^24^ applying ten 0.238 mm^2^ fields of view. Finally, Method 4–7 are novel approaches quantifying tumour buds across the whole slide or large regions of interest; methods only made feasible by the application of automated image analysis. Despite the fact that the ITBCC guidelines represent the first international effort to standardise manual TB count methodology, consistent reporting may still be affected by subjectivity, and inter- and intra-observer variability. Furthermore, manual quantification can be a time consuming process. To overcome these issues and standardise the quantification of TB we designed a fully automated ML based methodology capable of quantifying both the number and density of TB across specific regions of interest within the whole slide image.

It is interesting to note that in this study, TB quantified across the tumour core outperformed “field of view” methodologies, based on the ITBCC or published recommendations, when assessed against DSS prediction. Together with previous studies^13–17^, our results link intra-tumoural budding to a worse prognosis in MIBC. Interestingly, in this study, and in contrast with the majority of work in other cancer types, the number or density of TB within the invasive front of MIBC was not significantly associated with DSS. Additionally, we assessed the association between TB and a number of clinicopathological features (see Supplementary Table S2). We, like studies in a variety of other cancer types^16,17,23^, found a statistically significant positive association between TB and TNM staging (χ^2^ = 11.037, p = 0.00089) in MIBC.

### Image analysis for automated TB quantification

Despite the fact that most previous studies have evaluated TB using haematoxylin and eosin (H&E), recent reports have provided meaningful information about TB and disease progression using both immunohistochemistry (IHC)^33–36^ and immunofluorescence (IF)^37,38^. The use of cytokeratin-based immuno-labelling as a tumour mask clearly identifies TB even in cases that display a high density of peri-tumoural inflammatory infiltrate or reactive stromal cells. This fluorescence cytokeratin-aided distinction of TB allows for a more accurate recognition of TB when applying automated image analysis. However, the automated quantification of histopathological features may still be confounded by tissue artefact and necrotic debris. This is particularly true for methodology that employs object segmentation based on signal intensity thresholds or sole colour information, which can result in false positive and false negative detections. A further obstacle to overcome, when employing threshold based image analysis across patient cohorts, is inter- and intra-heterogeneity. The large inter- and intra-sample heterogeneity of the signal utilised to automatically segment tissue, for example tumour from stroma, may lead to inaccurate segmentation when run across large cohorts. Machine learning, and in particular convolutional neural network and context based random forest, was used to overcome the above limitations. By enabling the automatic definition of high-dimensional decisions based on colour and hierarchical texture information, the employed algorithms yield a precise quantification of true tumour bud objects against other false positive objects while ensuring robustness against variability in the visual appearance of tumour regions (see Supplementary Materials M4–M7). We believe that our methodology represents an advancement in automated image analysis compared to other such studies in the literature^39,40^ that quantify histopathological features based on object-based thresholding or sole colour-based analysis.

### Deep learning for automated TB analysis

**Figure 3:**
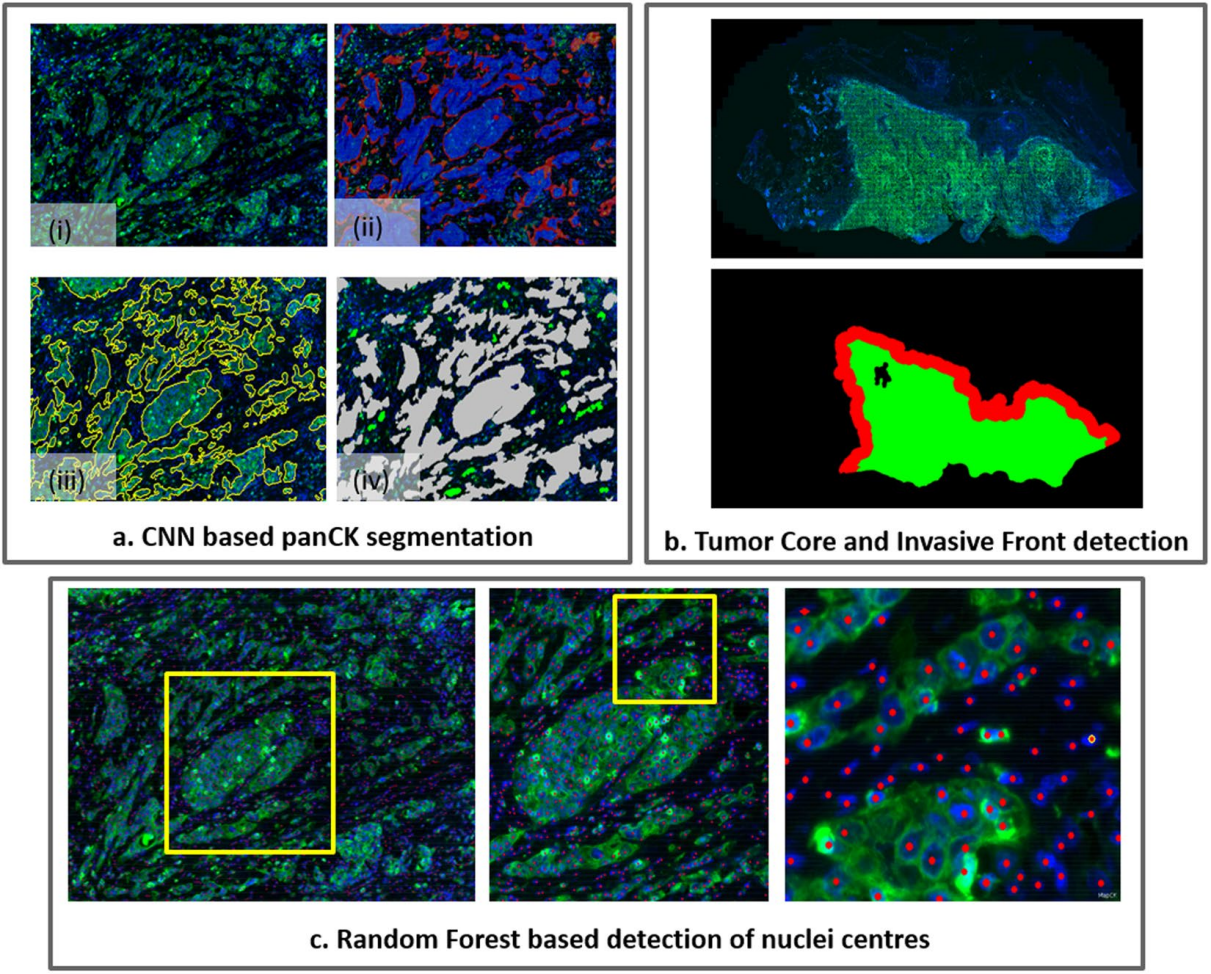
Three main components of image analysis. (**a**) CNN based segmentation of tumour regions: (i) PanCK and Hoechst channels; (ii) Initial detection of PanCK regions based on convolutional neural network-random forest model (blue) combined with additional detection based on Segnet (red); (iii) Segmentation result after local contour optimisation; (iv) Final detection and classification of tumour cell clusters, obtained from the tumour segmentation mask, in tumour buds (in green) or other tumour objects (in gray), based on the number of detected nuclei contained in each tumour cell cluster. (**b**) Automatic detection of the tumour core (green) and of the invasive front (in red) based on mathematical morphology applied to the tumour segmentation mask. (**c**) Results of the automatic detection of nuclei centres based on visual context regression random forest.

While our approach for tumour bud quantification builds on our own prior original and generic methods for IF image analysis^27,28^, Weis *et al*. recently proposed an application specific approach to recognise TB in IHC images of colorectal carcinoma based on classification convolution neural networks^41^. We believe that the two approaches differ in both their aim and their methodology. Regarding the methodology, Weis *et al*. performed segmentation of the tumour mask utilising mainly colour information: colour deconvolution was employed followed by k-mean clustering and heuristic post-processing rules. Tumour buds were then selected among the resulting tumour objects using a classification convolutional neural network. In contrast, we employ a semantic segmentation neural network for the segmentation of the tumour objects and detect all nuclei in the image. The selection of the tumour buds among the detected tumour objects then relies on the heuristic definition of a tumour bud based on the number of nuclei within a tumour cell cluster (see Fig. 3a.iv). In summary, while Weis *et al*.^41^ relies on colour information and heuristic rules to segment the tumour object and on deep learning for classification, we rely from the start on deep learning to segment the tumour objects and on the definition of tumour buds for their classification. Regarding the aim, we believe that utilising the explicit detection of nuclei and the explicit segmentation of tumour from stromal regions makes our approach more generic and usable in studies outwith specific tumour bud quantification. As an example, the explicit detection of nuclei further enables their classification into different sub-types and, in the context of immuno-oncology studies, opens the way towards the automated quantification of immune-related features^42^. In addition to enabling the automated estimation of the tumour core and invasive front, as presented in this study (see Fig. 3b), the explicit segmentation of tumour and stromal regions makes it further possible to evaluate the tumour infiltration level of the different immune cell types. We believe that relying on generic image analysis approaches is key for studying the joint impact of tumour buds and immune context, which would be an interesting extension of this work.

### Foreseen extension to IHC/H&E

Being trained on manual annotations performed on IF images, the aforementioned deep learning and random forest models for the segmentation of the epithelium regions and the detection of the nuclei location can at this stage only be applied for the analysis of IF images. However, by design, both algorithms are adaptable to staining used in routine practice (e.g. H&E or IHC) provided that corresponding manual annotations are available for training. As an example, we have first developed the regression random forest model used for cell detection for the analysis of H&E slides^28^, and have recently used convolutional neural networks for the segmentation of tumour region in the context of automated PD-L1 scoring^43^. The majority of whole slide scanning, in both the clinic and research laboratories, utilises brightfield illumination. As the algorithms described here can be adapted for brightfield scanned slides, the methodology allows the transition and uptake of these algorithms for the quantification of TB by facilities without fluorescence capability.

### Proposed TB stage model

Upon analysing the full MIBC cohort, no TB parameter was as significantly associated with patient survival as clinical TNM staging even though the parameter ‘number of TB in core’ was shown to be strongly prognostic for survival. By excluding the metastatic patients from the analysis, the objectively reported ‘number of TB in core’ (HR = 2.59, p = 0.01) outperformed all clinical features for predicting disease specific death (see Supplementary Table S1). Decision tree analysis of all the reported clinicopathological features combined with the seven tumour bud related features was performed to stratify patients into three survival groups, resulting in a novel TB stage model which utilises a combination of TNM staging and the ‘number of TB in core’ quantification. The model’s risk stratification of MIBC patients into three new staging groups improves upon the standalone clinical gold standard of TNM staging (see Table 3). While further research is required to validate these findings in larger cross-institutional studies, the current study provides evidence for the prognostic value of TB quantification.

### Conclusion

 This work demonstrates the automated detection and classification of tumour buds across digitised whole slide images which enables the quantification of TB in IF images. Based on this quantification, this study also reports for the first time that the presence of TB confers prognostic significance in the risk stratification of MIBC patients. In addition, a survival decision tree analysis was used to identify a combination of TB based features and clinical parameters, which, by separating non-metastatic MIBC patients into low and high TB groups, improves on current clinical gold TNM-based standard for prognosis.

## Methods

### Patients and specimens

All patients who underwent radical cystectomy for muscle-invasive bladder cancer in Edinburgh hospitals between the years 2006 to 2013, were collated into a study cohort from the hospital archives (N = 163). Due to the unavailability of patient material containing cancer tissue for some cases, the study number was reduced to 110. The archived formalin fixed paraffin embedded (FFPE) tissue block that contained the deepest invasion of cancer was selected for the 110 patients after review of haematoxylin and eosin (H&E) labelled slides by both a pathologist (DJH) and research scientist (PDC) (see Supplementary Figure S3). These samples were obtained from the NHS Lothian NRS BioResource Research Tissue Bank (Ethical status/approval ref: 10/S1402/33), conforming to protocols approved by East of Scotland Research Ethics Service (REC). Clinicopathological, treatment and survival data (see Table 1) was collected for each patient (see Supplementary Material M1). Disease specific survival was determined by a patient’s cause of death being attributed specifically to bladder cancer and patients were followed up for a total time of 113.13 months with a median survival time of 23.63 months The samples had been de-identified and unlinked from patient information prior to the commencement of the study. We excluded a further 10 out of 110 cases: 4 cases with unknown disease related death information and 6 cases with extensive tissue section artefacts. This resulted in a final and complete study cohort of 100 patients.

### Immunofluorescence and whole slide imaging

Automated immunofluorescence (IF) was performed on de-paraffinised 3 μm FFPE tissue sections using a Dako link 48 instrument (Dako, Agilent Technologies). The primary antibody against Pan-cytokeratin (PanCK; Cat# Z0622, Agilent Technologies) was utilised to visualise urothelial cells and nuclei were counterstained with Hoechst (Hoechst 33342, Cat# H3570, ThermoFisher Scientific). A Carl Zeiss AxioScan.Z1 whole slide scanner (Zeiss, Göttingen, Germany) was used to capture and digitise whole slide fluorescence images with a 20x objective (see detailed immunofluorescence and digitisation protocol in the Supplementary Material).

### Quantification of tumour buds through digital image analysis

The quantitative image analysis of tumour buds was performed using machine learning and deep learning approaches based on Definiens Tissue Phenomics® technology (Definiens AG, Munich, Germany)^44,45^. The automated image analysis algorithms were developed for the detection of nuclei and the segmentation of urothelial-specific PanCK positive regions from both stroma and artefacts prior to the classification and quantification of tumour buds. The following paragraphs provide more details on the image analysis methods.

### Detection of tumour cell clusters

The tumour cell clusters are defined as the connected components of the tumour mask (PanCK positive labelled cells). The tumour mask is segmented by ensemble prediction of two deep-learning models, trained on manual annotations. The two models read as follow. First, a semantic segmentation convolutional neural network (CNN) was developed based on the Inception architecture^46^ modified to include transposed convolution layers^47^ and skip connections^48^. As detailed in our previous work^27^, the second model combined a layer normalisation step, a fully CNN and a classification visual context Random Forest (RF)^49^. In comparison to our previous work^27^, the second model’s PanCK normalisation step relied on the detection of the PanCK reference objects from the output of the first network and not from heuristic rules. The outputs of the two networks were then ensemble by merging the two tumour masks detected by either of the two networks. The networks were trained based on the manual delineation of tumour and non-tumour regions (including tissue and staining artefacts as well as necrosis) on a subset of images prior to their application across the entire cohort of images. Examples of detected tumour masks are provided in Fig. 3a. In a final step, we formed the tumour cell clusters as the connected components of the tumour mask. The tumour core and invasive front, which are defined as the main tumour mass and as its border with a width of 1000 μm (500 μm inside and 500 μm outside of the border) respectively, were estimated from the segmented tumour mask. First, the main tumour mass was segmented using morphological closing on the tumour mask, which removes small interstices between the detected tumour cell clusters, and the resulting closed regions that were too small to correspond to the core removed. Once the tumour mass was classified, morphological dilation (500 μm) and erosion (500 μm) were then used to identify the outer and inner invasive front, respectively. An example of detected tumour core and invasive margins are provided in Fig. 3b.

### Nuclei detection

Nuclei were detected based on the training and application of two regression visual context RF models that respectively predict the pixel-wise spatial proximity to the closest nucleus as well as the size of the corresponding nucleus. As detailed in our previous work^28^, these two resulting prediction maps are inputs to a “local maxima” algorithm that outputs the position of the nuclei. This is illustrated in Fig. 3c.

### Quantitative evaluation

We performed a quantitative evaluation of the tumour segmentation and the nuclei detection algorithms on a set of 10 and 5 validation fields of view respectively, selected on whole slide images unused for training (see Supplementary Materials M4–M7). The quantitative evaluation of the detected tumour masks against manually annotated masks resulted in an overall Dice score of 0.86. In addition, a total of 5844 nuclei were manually annotated to enable the evaluation of the nuclei counts per consolidated tumour cell clusters, obtained by intersecting the detected and the true tumour cell clusters. The scatter plot between the true number and the estimated number of nuclei for each of the resulting 196 tumour cell clusters is illustrated in Supplementary Figure S2. A Pearson correlation of 99.3% is reported. Qualitative results of tumour segmentation and nuclei detection are illustrated in Supplementary Materials M2 and M3.

### Tumour bud classification

Post tumour segmentation and nuclei detection, we counted the number of nuclei within each tumour cell cluster. As specified in the recommendations for tumour bud reporting in colorectal cancer^11,12^, this yielded tumour cell clusters to be classified as tumour buds if containing between 1 and 4 nuclei (see Fig. 3a).

### Tumour budding quantification

Given the detected tumour buds and the estimated tumour core and invasive front, we quantified the number and density of both peri-tumoural (tumour buds within the invasive front) and intra-tumoural (tumour buds within the tumour core) detected budding. More precisely, the four following automated quantification methods were employed:

- ***Method 1*:** Across the ten 0.785 mm^2^ fields of view containing the highest number of detected tumour buds within the image.
- ***Method 2*:** Across a single 0.785 mm^2^ field of view which contained the highest number of detected tumour buds.
- ***Method 3:*** Similar to Method 1 but with a smaller 0.238 mm^2^ field of view.
- ***Method(s) 4–7:*** Quantification of the total number of tumour buds and their density in the detected tumour core and in the detected invasive front.

The first and third methods are based on the quantification methods previously described by Karamitopoulou E. *et al*.^21^. The size of the field of views of Method 1 and Method 2 was defined as recommended by the International Tumour Budding Consensus Conference (ITBCC) for the reporting of TB in colorectal cancer^12^. The third method was proposed by Lohneis P. *et al*.^24^. The fourth method explored other quantification approaches than the ones previously described in the literature and leads to an additional set of four tumour bud related features. The resultant (m = 7) TB related features are set out in Table 2. Note that a larger set of features could easily be derived from the image analysis results. However, given the number of patients (N = 100), we followed the recommendations by Field *et al*.^50^ and restricted in this study the number of tested tumour bud related predictors. This aimed at limiting the impact of multiple hypothesis testing.

### Image analysis infrastructure

The convolutional neural networks were trained and predicted using the open source Python deep learning library Keras (https://keras.io/) (Version 2.0.9) running on top of TensorFlow^51^ (Version 1.4.0). Other operations, such as the training and prediction of the classification and regression RF models as well as the computation and export of the tumour bud related features, were performed in an extended version of the Definiens Developer XD software^52^.

### Univariate statistical analysis

Together with the seven TB related features described in the previous section, we included the following clinicopathological features for univariate statistical analysis on all MIBC patients: TNM stage, pT stage, lymph node status, presence of metastasis, grade, growth pattern, treatment option, age and gender. Univariate Log rank test and cox regression analysis were performed in R (Version 3.3.2) using the survival package (Version 2.39–5) and the survminer package (Version 0.4.3). When meaningful, we performed p values correction to account for the m = 7 hypotheses resulting from the definition of the TB related features. Corrected p values, or q values, were computed using the R package Bioconductor^53^. Because we did not have enough p values to warrant the estimation of the overall proportion of true null p values π_0_, the input parameter λ was set to 0, which resulted in following the most conservative Benjamini methodology.

### Survival decision tree for multivariate statistical analysis

Our survival decision tree (see Fig. 1) analysis reads as follows: starting with a root consisting of all MIBC patients (N = 100), we iteratively identified the best decision (i.e. the best pair of feature and associated cut-off) among all the input features and all possible cut-off values which maximised the difference in the survival distributions of the two resulting sub-groups. This difference was quantified by log rank p value with a minimum prevalence of the two resulting low/high groups of 25%. Since the two clinical features of growth pattern and treatment combination are strictly categorical, no cut-off can be applied for splitting. In these two cases, the split is instead defined by patients having the given growth pattern versus patients having a different growth pattern, or by patients having received the given treatment combination versus patients having received a different treatment. The optimisation and leave-one-out cross validation of the decisions were developed in Python (Version 3.5.3) using the lifelines package (version 0.14.3). In the next paragraphs, we further justify the use of a survival decision tree vs. a standard classification tree, detail the employed leave-one-out cross-validation methodology, report our leaf merging strategy and finally describe the statistical analysis performed for comparison of the resulting TB stage model against the standard TNM stage model.

#### Survival decision function

Our survival decision tree differs from the standard classification tree: while the standard classification tree finds at each node the decision that minimises the misclassification rate between the two resulting branches, we instead return the decision that minimises the log rank test p value between the survival characteristics of the two resulting branches. By bypassing the need of an arbitrary set of classes, the proposed survival decision tree has the advantages of keeping both the time and event information and of maintaining the continuous characteristic of survival information. In contrast, the use of a classification tree would require to arbitrarily define the classes either (1) by directly taking the event (death/survival) as class, or (2) by classifying patients with time-to-event higher than an arbitrary reference time (e.g. median survival time) as high survival class and patients with time-to-event lower than this reference time as low survival class.

#### Leave-one-out cross validation

Each split’s feature and associated optimal cut-off value were validated together with the whole decision tree using leave-one-out pre-validation to prevent overfitting^54^, resulting in the decisions at each node to be independently optimised for each patient. While overfitting the decisions on all patients would result in stronger separation between the survival groups, the employed leave-one-out approach enables a better assessment of how the found results would generalise to another dataset. This is of high importance given the limited size of our current dataset (N = 100). The leave-one out analysis reads as follows: for a given patient, the decision is chosen to minimize the log rank p value of the separation of the remaining N-1 patients into low and high survival branches. Each patient is therefore left out of the selection of its optimal decision. The final grouping is obtained by applying the individual leave-one-out cross validated decisions to the corresponding individual patients, which results in the independence between the patients and the decisions.

#### Leaf merging

Because the low number of TNM IIIB patients (4%, see Table 1) would not result in any meaningful statistical observation, we focused the analysis on the standard TNM stages II, III and IV for MIBC patients. To ensure comparability with this TNM staging, we enforced the proposed survival decision tree to output three final categories by setting the maximum depth to two and by splitting only the most populated node at depth one.

#### Statistical tests

Categorical Cox regression analysis was finally performed on the three standard TNM groups (II, III, and IV) as well as on the three categories (II’, III’, IV) resulting from the proposed survival decision tree model, also denoted ‘TB stage model’. The hazard ratios together with their confidence intervals were computed to estimate the effect of the two respective staging methods. The respective TNM stage II and TB model stage II’ were taken as baseline for both models, leading to the following analysis: TNM III vs TNM II, TNM IV vs TNM II, TB III’ vs TB II’ and TB IV vs. TB II’. Overall significance of the two models were evaluated with the likelihood ratio test, Wald test and score log-rank test. This last analysis was performed together with the computation of the associated Kaplan Meier curves in R (Version 3.3.2) using the survival package (Version 2.39–5) and the survminer package (Version 0.4.3).

### Ethical approval and informed consent

Ethical approval for this study was obtained from the NHS Lothian Tissue Governance Unit (Ethical status/approval ref: 10/S1402/33), conforming to protocols approved by East of Scotland Research Ethics Service (REC). Patient material was surplus to diagnosis and no further consent was required.

## Supplementary information

Supplementary Information

Supplementary Material 1

Supplementary Material M2

Supplementary Material M3

Supplementary Material M4

Supplementary Material M5

Supplementary Material M6

Supplementary Material M7

## Data Availability

To enable the replication of the statistical analysis and results, the computed tumour budding features are available for each patient together with the corresponding survival information and clinical features (see Supplementary Material M1). We also provide the reader with the tiff images corresponding to fields of view used for validation of the segmentation and detection algorithm (see Supplementary Materials M4–M7). The current cohort being still under investigation, the raw image data is not available at this stage. The software platform Definiens Developer XD is commercially available.
