# Supplementary material for: Automated tumour budding quantification by machine learning augments TNM staging in muscle-invasive bladder cancer prognosis
Source: Sci Rep. 2019 Mar 26;9:5174. doi: 10.1038/s41598-019-41595-2 (PMC6435679; doi:10.1038/s41598-019-41595-2)
Supplement: Supplementary file 1 — Supplementary Information [file 41598_2019_41595_MOESM1_ESM.docx]

**Title**: Automated tumour budding quantification by machine learning augments TNM staging in muscle-invasive bladder cancer prognosis.

**Authors:** Brieu Nicolas^1^, Gavriel G Christos^2^, Nearchou P Ines^2^, Harrison J David^2^, Schmidt Günter^1^ and Caie D Peter^2^*

^1^Definiens AG, Bernhard-Wicki-Straße 5, 80636 München, Germany.

^2^School of Medicine, University of St Andrews, North Haugh, St Andrews, Fife, KY16 9TF, UK.

Brieu Nicolas and Gavriel G Christos contributed equally to this work.

**Supplementary Information**

**Tissue section preparation.** Paraffin sections (3μm) from FFPE tissue blocks of MIBC, containing whole tissue were mounted on positive charged microscope slides and incubated overnight in a 37°C oven. Following overnight incubation, sections were dewaxed three times for five minutes each with xylene and rehydrated in decreasing concentrations of ethanol for 2 minutes each (100% x 2, 80% and 50%) and finally running water for 2 minutes per wash.

**Immunofluorescence.** Post tissue section preparation heat induced antigen retrieval was executed in a pressure cooker. The antigen retrieval buffer was consisting of 0.15mM Sodium Citrate pH6 and pre-boiled in a microwave for 12 minutes. Tissue slides were then placed in the pre-heated pressure cooker and microwaved for an additional 5 minutes. Slides were left to cool down by adding cold water in the heated buffer and washed in 0,1% PBST (Phosphate saline solution / 0.1% Tween) for 5 minutes. Following antigen retrieval, 3% hydrogen peroxide solution (3% H_2_O_2_) was used to wash the slides for 5 minutes and therefore block endogenous hydrogen peroxidase. Slides were washed again in 0.1% PBST for 5 minutes and incubated in Dako serum free protein block (Dako, #X0909) for 10 minutes to prevent non-specific antibody binding. Sections were next incubated in a primary antibody solution diluted in Dako antibody diluent (Dako, #S0809) at either room temperature for 30 minutes or overnight at 4°C. Supplementary Table S3 comprises information on the primary antibodies that were used during the experiments.

**Immunofluorescence Visualisation.** 5-plex immunofluorescence was performed by incubating slides in prediluted HRP conjugated secondary antibody (Dako, Envision anti-Rabbit HRP #4003 or anti-Mouse HRP #4001) for 30 minutes in the dark at room temperature and washed three times for 5 minutes each in 0.1% PBST. Tyramide Signal Amplification (TSA) Cy5 (Perkin Elmer, Cyanine 5 Amplification Reagent, #FP1171) was diluted (1:100) in target signal amplification diluent (Perkin Elmer, #FP1498) and utilised to incubate the tissue for 10 minutes in the dark at room temperature. The slides were washed three times for 5 minutes each in 0.1% PBST prior the first microwave stripping and blocking step (same procedure with antigen retrieval and endogenous blocking as previously described. The only difference is that the slides are heated in the microwave for 17 minutes in defrosting mode instead of 5 minutes after the initial pre-heated step of the antigen retrieval buffer). For the second primary antibody incubation and visualisation step, the slides were incubated in a primary antibody solution diluted in Dako antibody diluent (Dako, #S0809) at either room temperature for 30 minutes or overnight at 4°C and washed three times for 5 minutes each in 0.1% PBST. The slides were then incubated in pre-diluted HRP conjugated secondary antibody (Dako, Envision anti-Rabbit HRP #?4003 or anti-Mouse HRP #4001) for 30 minutes in the dark at room temperature and washed three times for 5 minutes each in 0.1% PBST. TSA FITC (Perkin Elmer, Fluorescein Amplification Reagent, #FP1168) was diluted (1:100) in target signal amplification diluent (Perkin Elmer, #FP1498) and used to incubate the tissue for 10minutes in the dark at room temperature. The slides were washed three times for 5 minutes each in 0.1% PBST prior the second microwave stripping and blocking step (as previously described). For the third and fourth primary antibody incubation and visualisation step, the slides were incubated in two primary antibody solutions diluted in Dako antibody diluent (Dako, #S0809) at either room temperature for 30 minutes or overnight at 4°C and washed three times for 5 minutes each in 0.1% PBST. Anti-rabbit biotinylated antibody (Perkin Elmer, IgG Goat Biotin-labeled, # NEF813001EA) was diluted (1:250) in pre-diluted HRP conjugated secondary antibody (Dako, Envision anti-Rabbit HRP #4003 or anti-Mouse HRP #4001) and used to incubate the tissue for 30 minutes in the dark at room temperature. The slides were next washed three times for 5 minutes each in 0.1% PBST. Alexa Fluor 750 conjugated streptavidin antibody (Thermo Fisher Scientific, #S21384) was diluted (1:50) in Dako antibody diluent (Dako, #S0809) and utilised to incubate the tissue for 30 minutes in the dark at room temperature. Lastly, the slides were washed three times for 5 minutes each in 0.1% PBST. For the counterstaining and mounting step, Hoechst 3342 (Thermo Fisher Scientific, #H3570) was diluted (1:20) in deionised water and used to incubate the tissue in the dark at room temperature for 30 minutes. The slides then were washed three times for 5 minutes each in 0.1% PBS and dehydrated in 80% ethanol for 1minute prior air-dried, mounted in prolong gold antifade mounting medium (Thermo Fisher Scientific, #P36930) and coverslipped.

**Imaging Acquisition.** Whole slide images were captured using Carl Zeiss AxioScan.Z1 whole slide scanner (Zeiss, Göttingen, Germany). The fluorescent filters were carefully selected to minimize ‘bleed through’ from fluorophores with overlapping excitation and emission spectra. The fluorescent filters and cubes were purchased from Zeiss (Göttingen, Germany) and Laser 2000 (Cambridgeshire, UK) and comprised the following filter sets: Zeiss filter set 49 for Hoechst or equivalent, 38HE for FITC or equivalent, 48HE for Cy3 or equivalent, 50 for Cy5 or equivalent and Semrock CY7-B-ZHE BrightLine single-band filter set mounted in a cube, optimized for Cy7 and other like fluorophores. The following exposure times were set on the AxioScan.Z1 whole slide scanner: 10ms for Hoechst, 10ms for FITC, 10ms for Cy3, 10ms for Cy5 and 600ms for Cy7. Colibri.2 LED light source was used for excitation less than 700nm while HXP metal halide light source was used for excitation over 700nm. ZEN imaging software (Blue Edition, 2.3 Lite) was utilised for editing and viewing the captured images allowing the navigation to the regions of interest and comparison between the virtual slides at 4x, 10x and 20x objectives. The CZI file format from Zeiss was processed and exported for image analysis.

*For the purpose of this study, only Pan-cytokeratin was utilised in the analysis reported here. CD3, CD8 and PD-L1 markers are aligned with a separate study outwith of this project’s scope.

**Supplementary Figure Legends**

**Supplementary Figure S1.** Kaplan Meier plot and risk table of disease specific survival for MIBC patients in dependence of (a) grade, (b) treatment option (cf. supplementary table S4), and (c) growth pattern. Note that in (b) and (c), groups containing less than 5 patients are not displayed not to hamper the clarity of the figures.

**Supplementary Figure S2.** Scatter plot between the true number of manually annotated nuclei and the estimated number of detected nuclei in each of the intersections between the true tumour objects and the detected tumour objects. Both axis are logarithmic.

**Supplementary Figure S3.** Tumour Budding in H&E images. **a.** Intratumoural budding in lower magnification (0.50x). The box indicates the magnified region of interest that is visualised at higher magnification in part b. **b.** Intratumoural budding in higher magnification (20x). Tumour buds are indicated by the arrows. **c**. Peritumoural budding in lower magnification (0.50x). The box indicates the magnified region of interest that is visualised at higher magnification in part d. **d.** Peritumoural budding in higher magnification (20x). Tumour buds are indicated by the arrows.

**Supplementary Tables**

| Supplementary Table S1. Results of univariate log rank test and univariate Cox regression analysis of clinical and image analysis features associated with disease specific survival in MIBC patients without metastasis (Stage II and III) (N=70). Reported p-values and hazard ratios are obtained for each feature through leave-one-out pre-validation of the optimal separation cut-off used to separate the patients into two optimal low/high sub-groups. (***) indicates p<0.001, (**) p<0.01 and (*) p<0.05. For the seven tumour budding features, p-values corrected for the multiple tumour budding hypothesis (q-values) are reported together with the original p-values. | | | |
| --- | --- | --- | --- |
| Features | **Log rank test** | **Cox regression** | |
|  | **p-value / q-value** | **Hazard Ratio** | **p-value / q-value** |
| TNM stage | 0.1103 | 1.97 [0.84 4.63] | 0.1172 |
| pT stage | 0.0254 * | 2.26 [1.08 4.71] | 0.0296 * |
| Lymph node status | 0.2012 | 1.73 [0.73 4.06] | 0.2068 |
| Metastasis |  | - | - |
| Grade | 0.2294 | 2.35 [0.59 9.90] | 0.2435 |
| Growth Pattern | 0.4071 | 0.61 [0.18 2.00] | 0.4119 |
| Treatment | 0.0494 * | 0.17 [0.02 1.26] | 0.0832 |
| Gender | 0.6452 | 0.84 [0.40 1.75] | 0.6456 |
| Age | 0.5688 | 0.80 [0.37 1.70] | 0.5695 |
| Number of TB in core | **0.0091 ** / 0.0637** | **2.59 [1.23 5.47]** | **0.0119 * / 0.0833** |
| Number of TB in invasive front | 0.6427 / 0.8443 | 0.84 [0.40 1.7 ] | 0.6431 / 0.8445 |
| Density of TB in core | 0.9324 / 0.9324 | 0.96 [0.46 2.02] | 0.9324 / 0.9324 |
| Density of TB in invasive front | 0.1122 / 0.2977 | 1.91 [0.84 4.32] | 0.1185 / 0.3178 |
| Number of TB in a single 0.785mm2 field of view | 0.6641 / 0.8443 | 1.19 [0.54 2.61] | 0.6645 / 0.8446 |
| Number of TB in ten 0.785mm2 fields of view | 0.1276 / 0.2977 | 2.08 [0.79 5.45] | 0.1362 / 0.3178 |
| Number of TB in ten 0.238mm2 fields of view | 0.7237 / 0.8443 | 1.14 [0.53 2.47] | 0.7239 / 0.8446 |

| **Supplementary Table S2.** Association of the ‘number of TB in core’ image analysis feature with the clinicopathological data. | | | |
| --- | --- | --- | --- |
|  | **Pearson’s Chi-squared test** | | |
| **Features** | **χ2** | **Degrees of freedom** | **p- value** |
|  |  |  |  |
| **TNM staging (II,IIIA,IIIB / IV)** | **11.037** | **1** | **0.00089** |
| pT stage (2a,2b,3a,3b / 4a,4b) | 7.065 | 1 | 0.0078 |
| Lymph node status (N0 / N1,N2) | 2.52 | 1 | 0.11 |
| Gender (male / female) | 0.46 | 1 | 0.49 |
| Age (<71 / >=71) | 0.13 | 1 | 0.71 |
| Treatment (Mitomycin C: Yes/No) | 0.0044 | 1 | 0.94 |
| Growth Pattern (Papillary/Other) | 1.16 | 1 | 0.28 |
| Grade (G2, G2-3, G3) | 0.47 | 1 | 0.67 |

| **Supplementary Table S3.** Primary antibodies used for immunofluorescence. | | | | |
| --- | --- | --- | --- | --- |
| **Antibody** | **Supplier** | **Catalogue Number** | **Species** | **Dilution** |
| PanCK | Agilent Technologies | Z0622 | Rabbit-Polyclonal | 1:100 |
| CD3 | Agilent Technologies | A0452 | Rabbit-Polyclonal | 1:400 |
| CD8 | Agilent Technologies | M7103-Clone C8/144B | Mouse-Monoclonal | 1:200 |
| PD-L1 | Cell Signaling Technology | EIL3N-13684S | Rabbit-Monoclonal | 1:100 |

| **Supplementary Table S4.** Set of treatment options observed in the patient cohort having a prevalence higher than 5%. N/D: Non disclosed. | | | | |
| --- | --- | --- | --- | --- |
| **Treatment ID (% of patients)** | **Mitomycin C** | **BCG (ever)** | **Chemotherapy (ever)** | **Radiotherapy (ever)** |
| #1 (11%) | Yes | No | No | No |
| #2 (9%) | N/D | Yes | No | No |
| #3 (13%) | N/D | No | Yes | No |
| #4 (5%) | Yes | No | Yes | No |
| #5 (50%) | N/D | No | No | No |

| **Supplementary Table S5.** Association of the proposed TB stage model with the clinical TNM model (left) and the Grade model (right). Chi-square analysis reported (Χ2=4.28, df=4, p=0.37) between the TB stage model and the Grade. | | | | | | |
| --- | --- | --- | --- | --- | --- | --- |
| **TB stage model** | **TNM II** | **TNM III** | **TNM IV** | **G2** | **G2-3** | **G3** |
| II’ | 24 | 27 | 0 | 2 | 1 | 22 |
| III’ | 1 | 18 | 0 | 6 | 0 | 39 |
| IV | 0 | 0 | 30 | 3 | 1 | 26 |

| **Supplementary Table S6.** Association of the proposed TB stage model with the Growth Pattern. Chi-square analysis reported (Χ2=8.84, df=8, p=0.3564) between the TB stage model and the Growth Pattern. | | | | |
| --- | --- | --- | --- | --- |
| **TB stage model** | **Solid** | **Solid/Papillary** | **Papillary** | **Other (small cell & sheet)** |
| II’ | 31 | 8 | 10 | 2 |
| III’ | 14 | 4 | 1 | 0 |
| IV | 25 | 4 | 1 | 0 |

**Supplementary Figures**


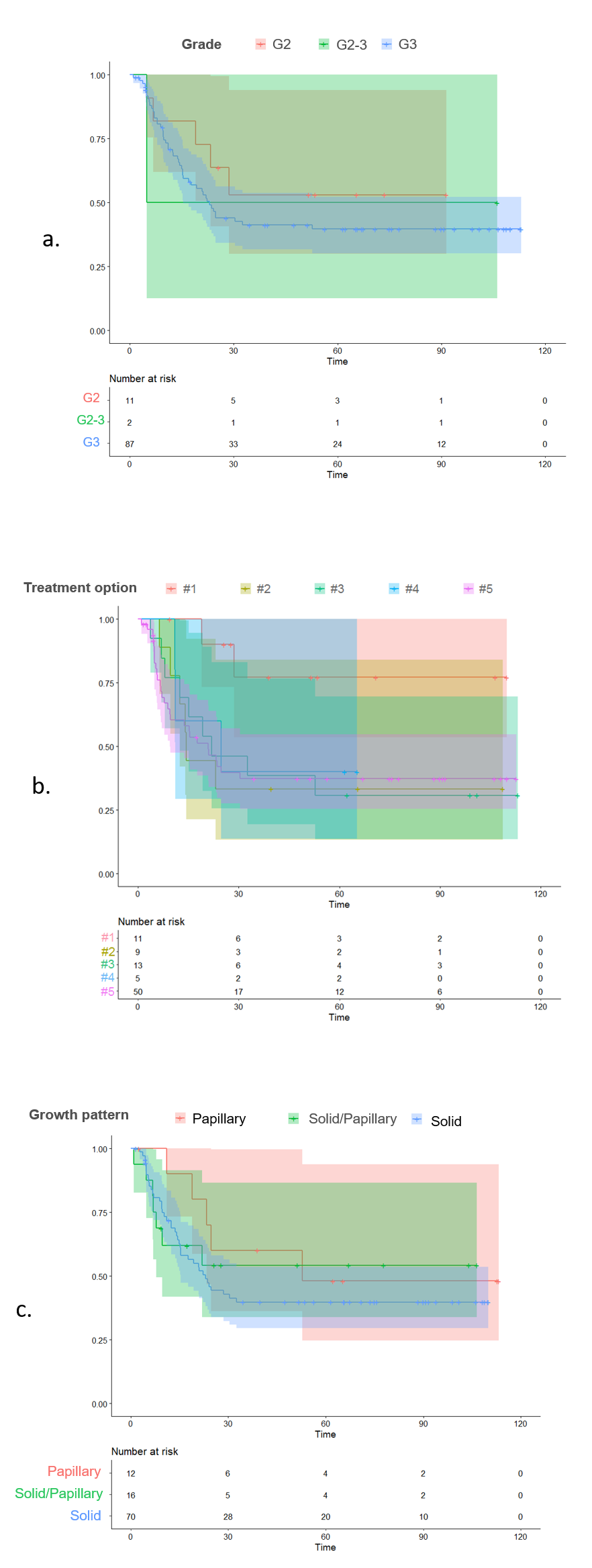


**Supplementary Figure S1**


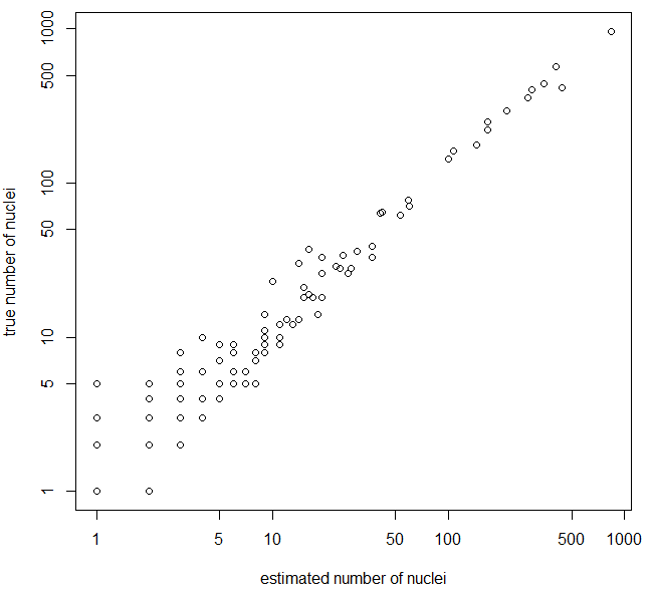


**Supplementary Figure S2**


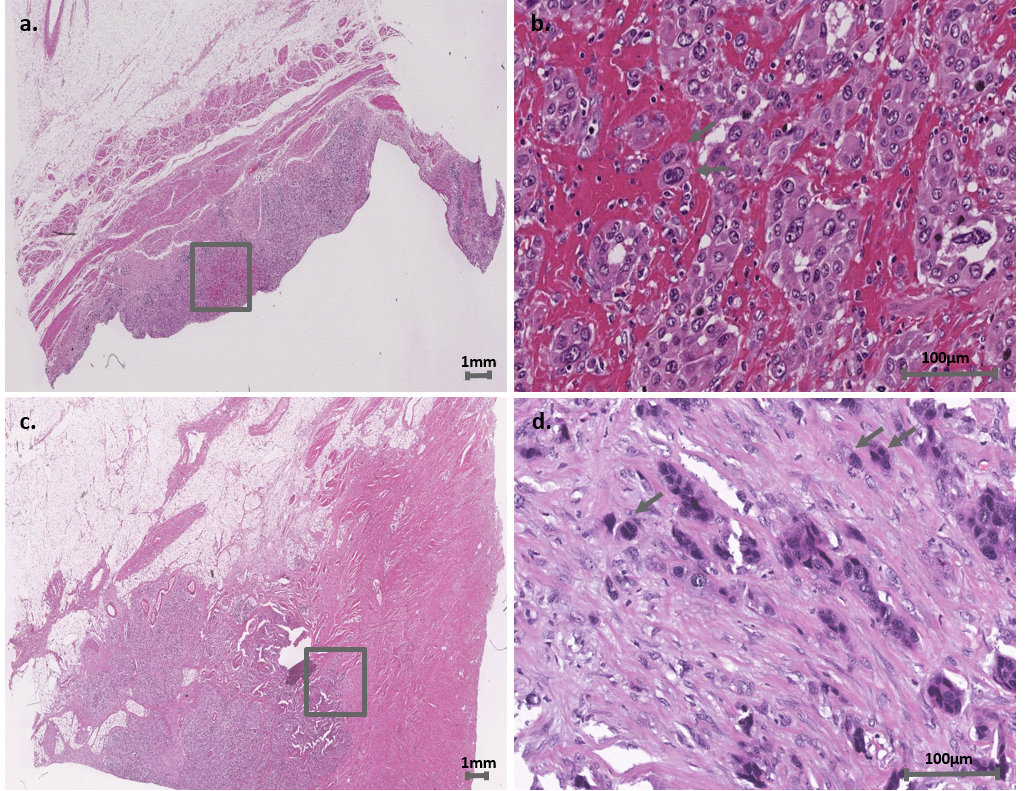


**Supplementary Figure S3**
