# Supplementary material for: Automated tumour budding quantification by machine learning augments TNM staging in muscle-invasive bladder cancer prognosis
Source: Sci Rep. 2019 Mar 26;9:5174. doi: 10.1038/s41598-019-41595-2 (PMC6435679; doi:10.1038/s41598-019-41595-2)
Supplement: Supplementary file 3 — Supplementary Material M2 [file 41598_2019_41595_MOESM3_ESM.pdf]

Title: Automated tumour budding quantification by machine learning augments TNM staging in muscle-invasive bladder cancer prognosis.

Authors: Brieu Nicolas[1], Gavriel G Christos[2], Nearchou P Ines[2], Harrison J David[2], Schmidt Günter[1] and Caie D Peter[2]*

[1]Definiens AG, Bernhard-Wicki-Straße 5, 80636 München, Germany.

[2]School of Medicine, University of St Andrews, North Haugh, St Andrews, Fife, KY16 9TF, UK.

Brieu Nicolas and Gavriel G Christos contributed equally to this work.

Supplementary Material M2. Qualitative results of the tumour mask segmentation algorithm, on the 10 FOVs provided in supplementary materials M2-M7. True positive pixels are in green, false positive pixels in yellow and false negative pixels in red. Note how artefacts are automatically removed from the analysis by the segmentation algorithm.

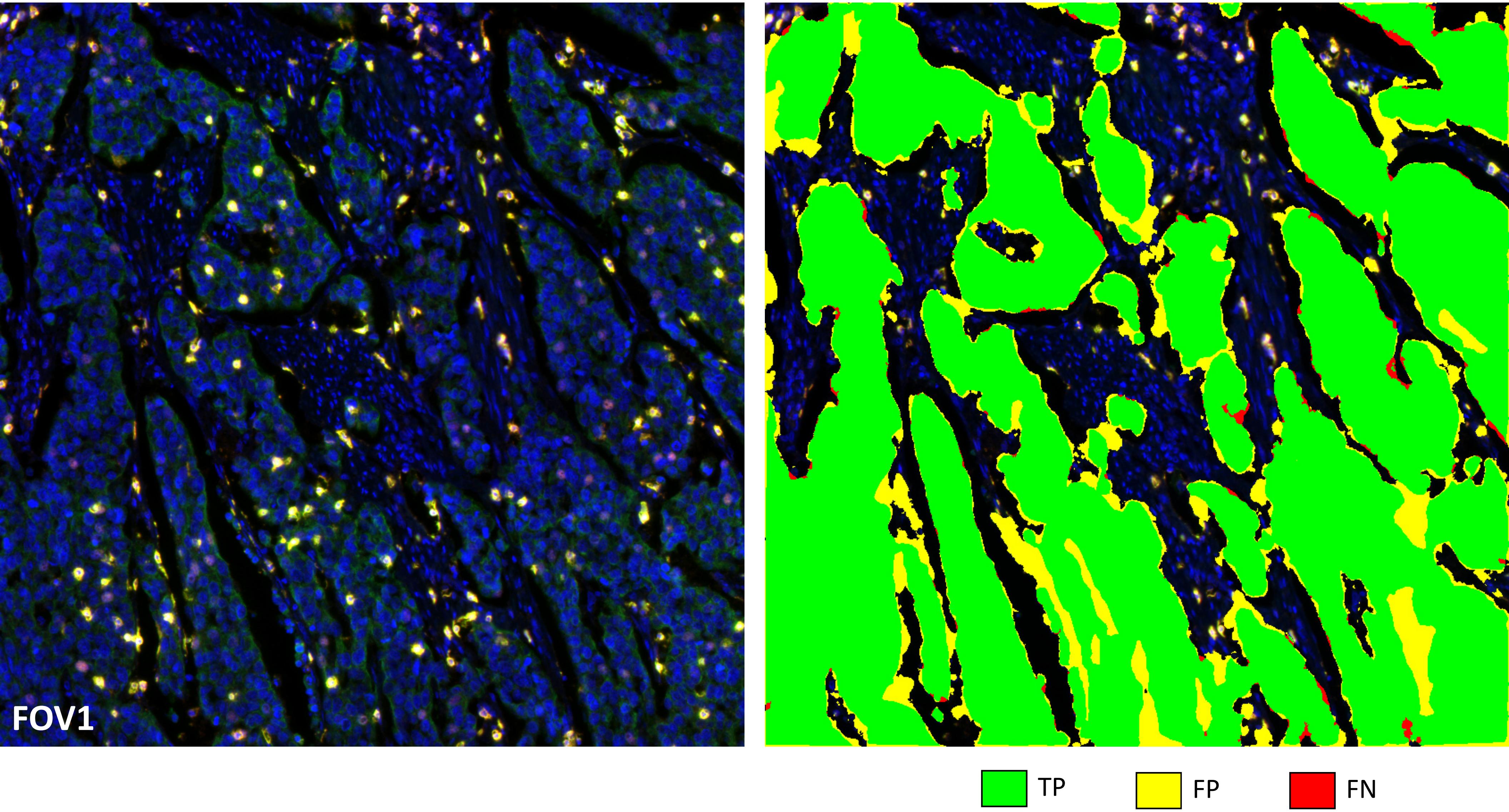
FOV1
TP
FP
FN

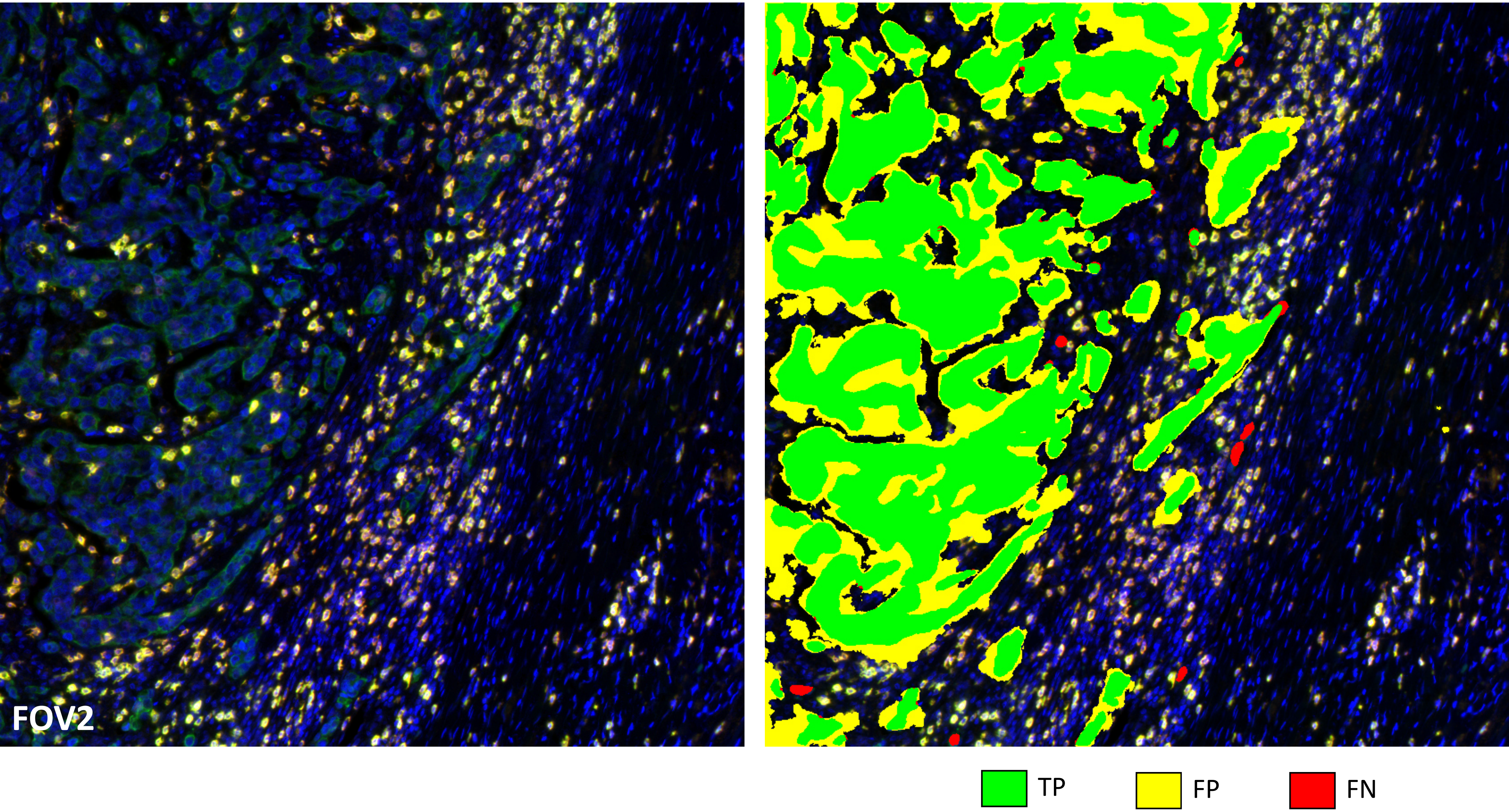
FOV2
TP
FP
FN

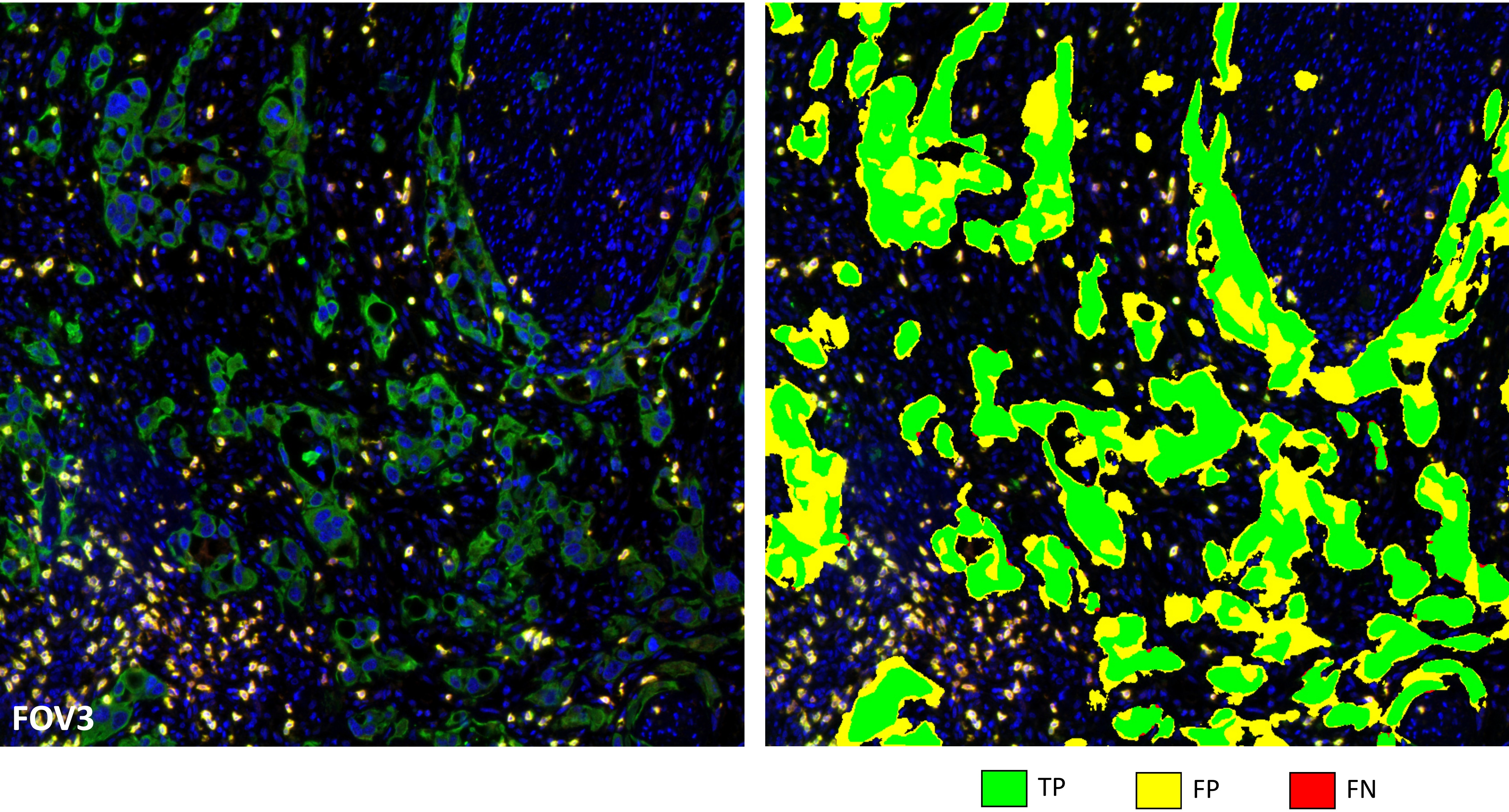
FOV3
TP
FP
FN

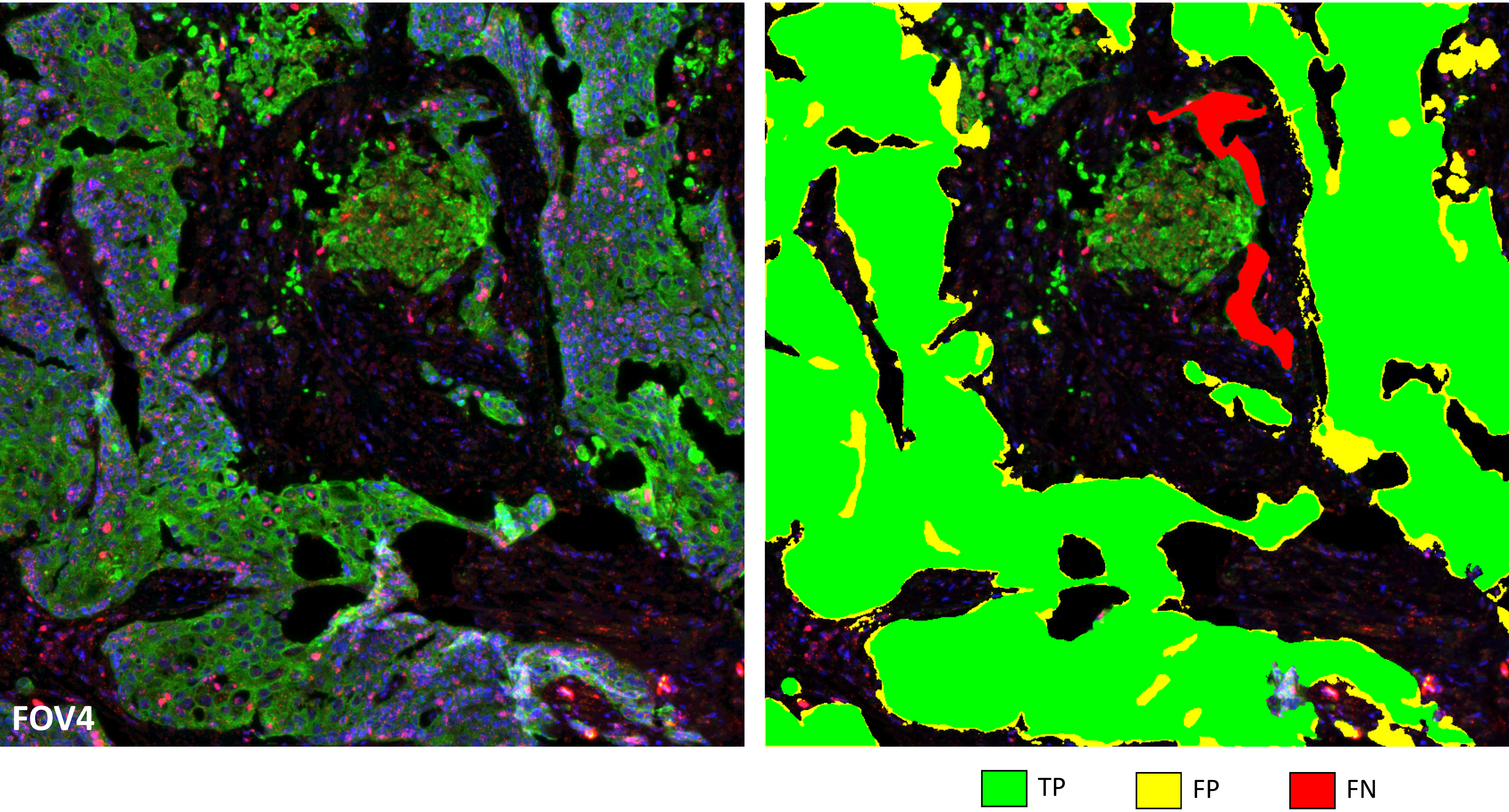
FOV4
TP
FP
FN

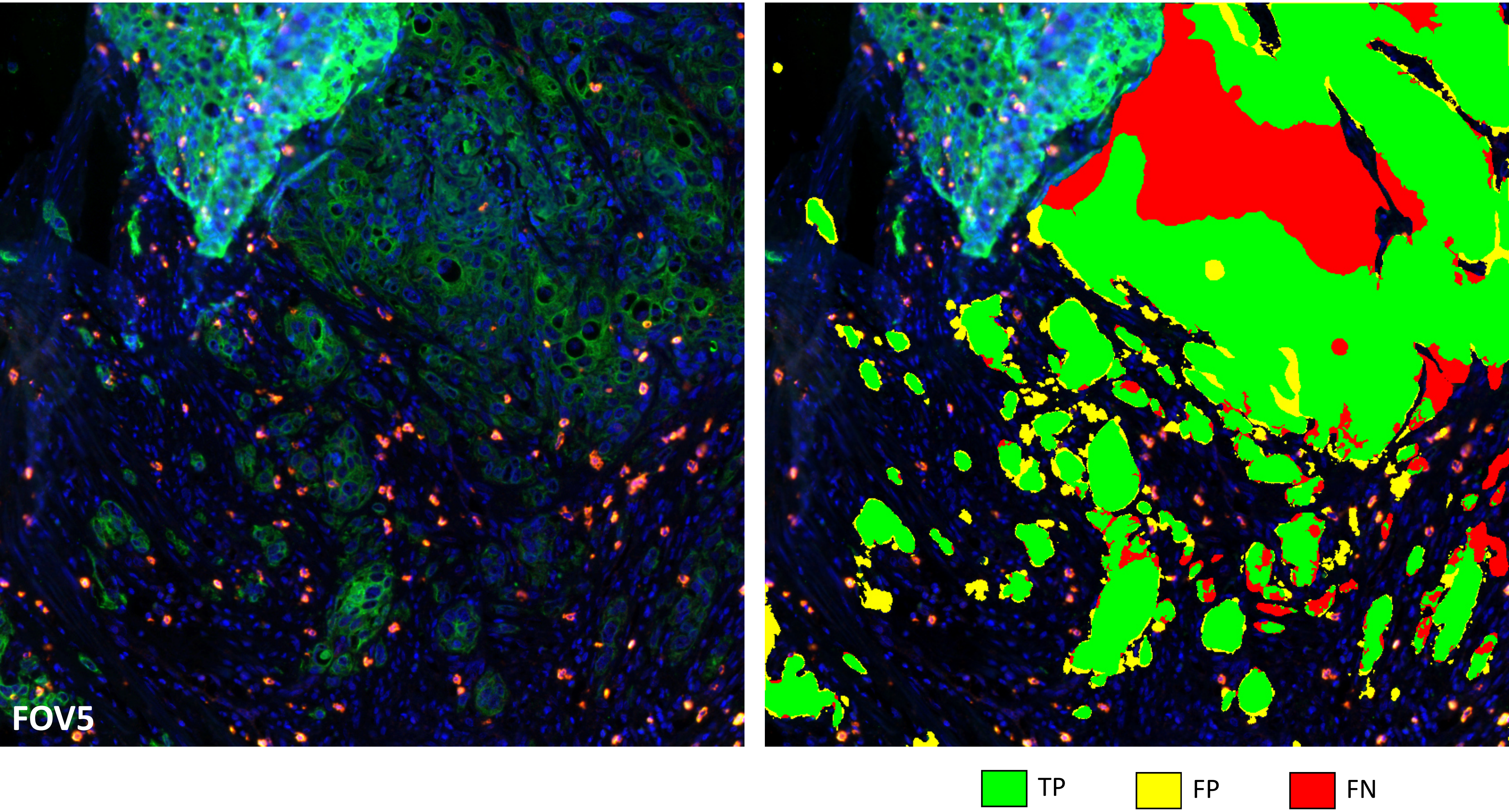

FOV5
TP
FP
FN

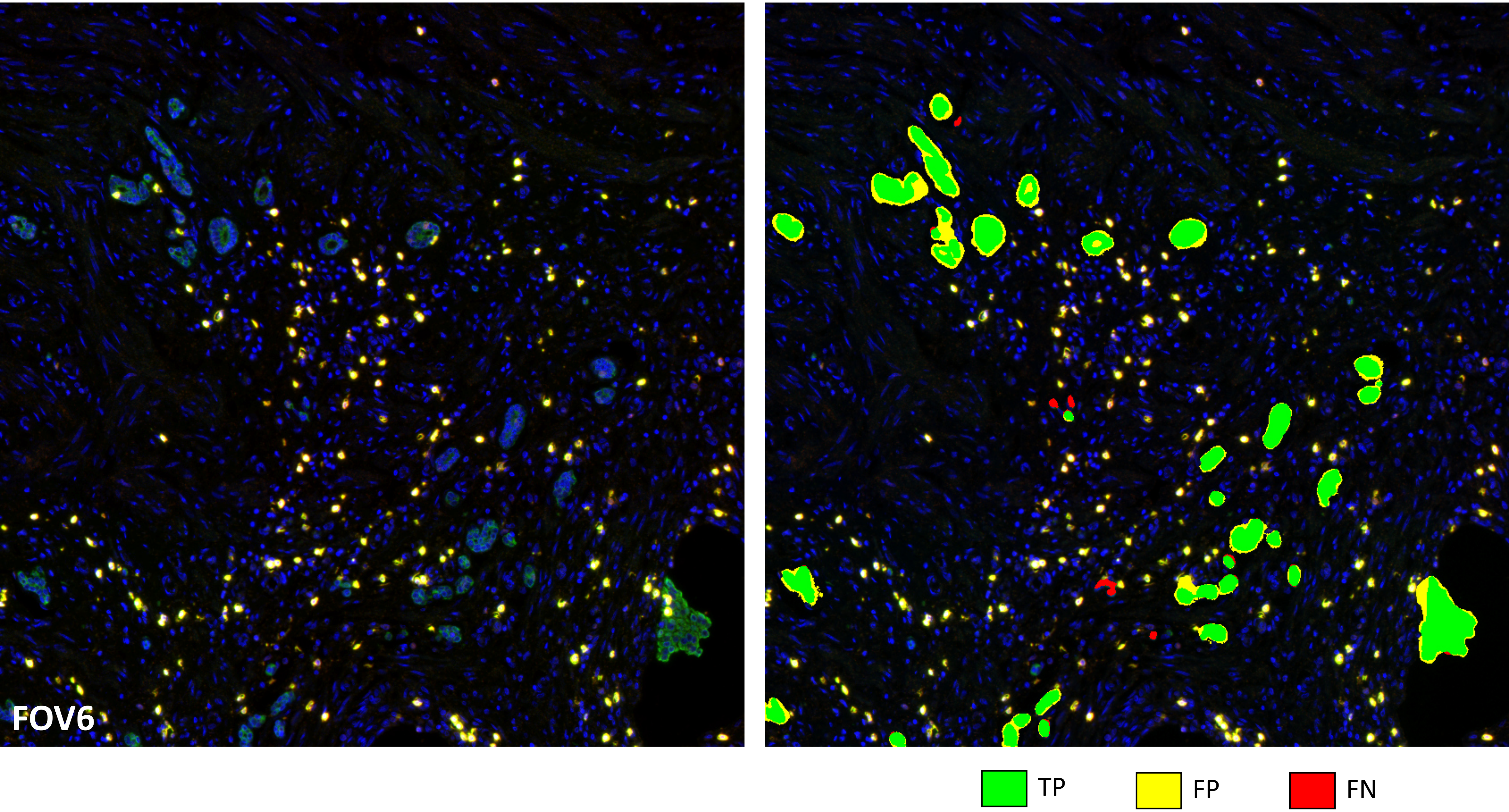
FOV6
TP
FP
FN

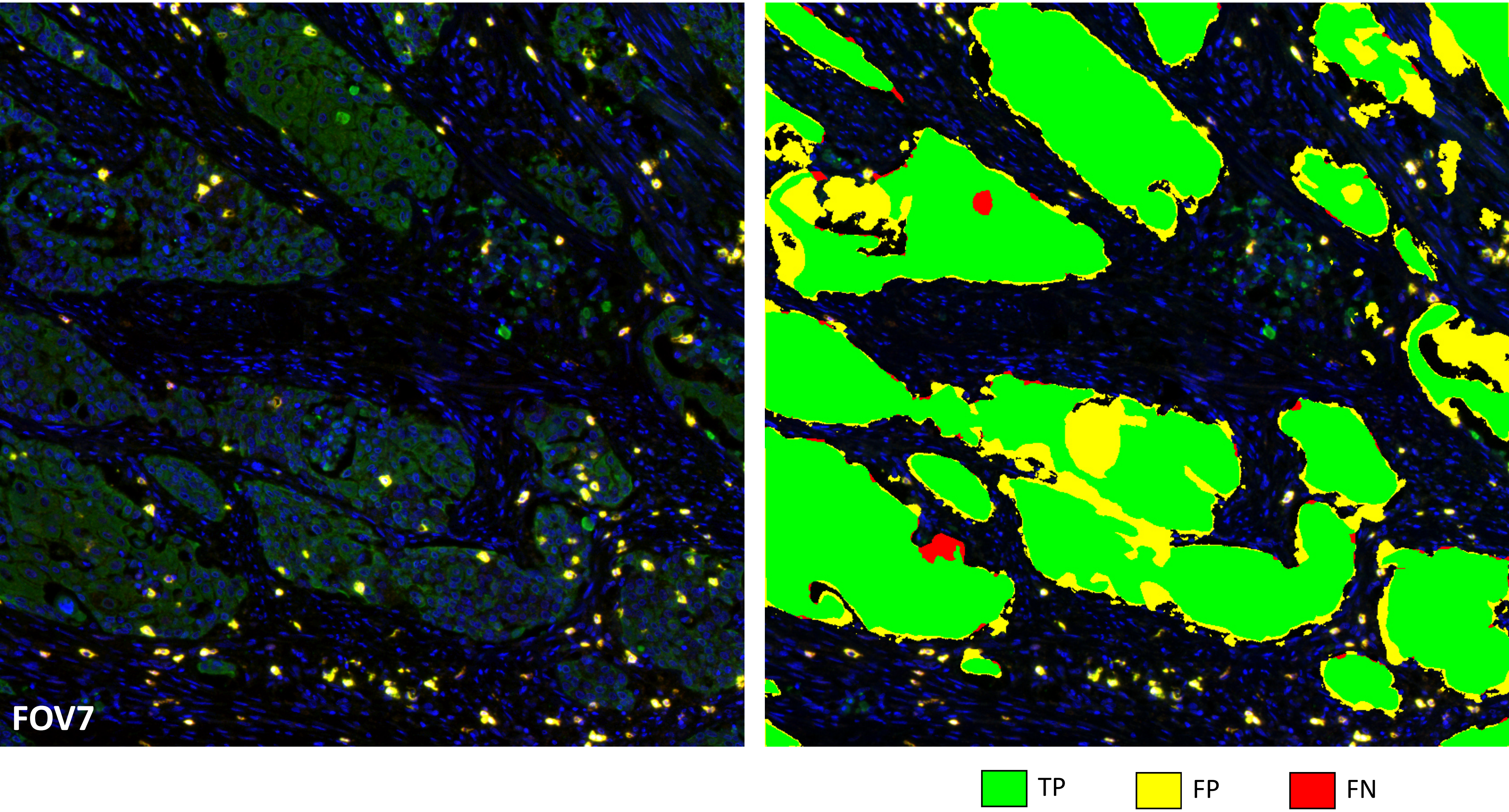
FOV7
TP
FP
FN

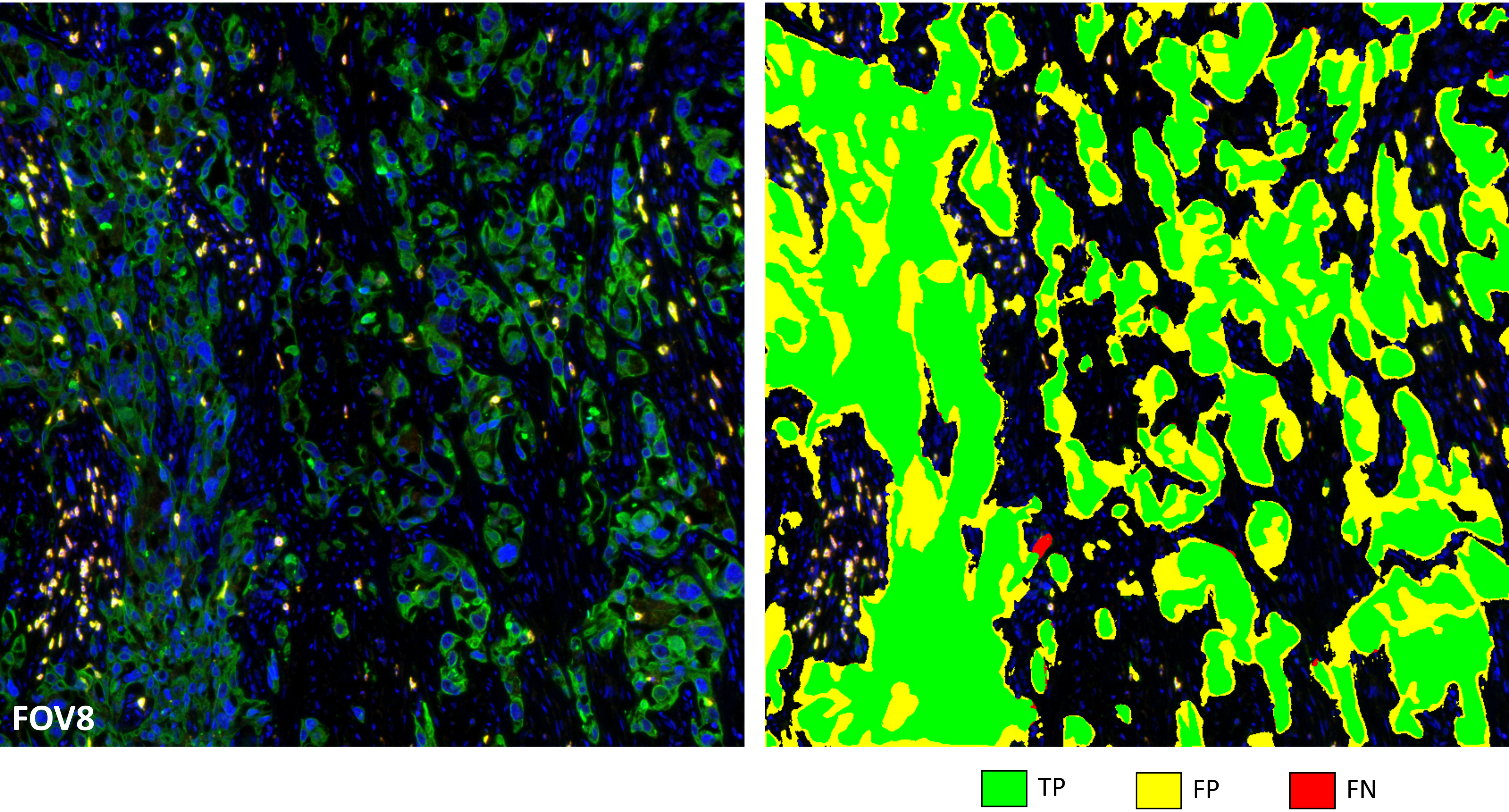
FOV8
TP
FP
FN

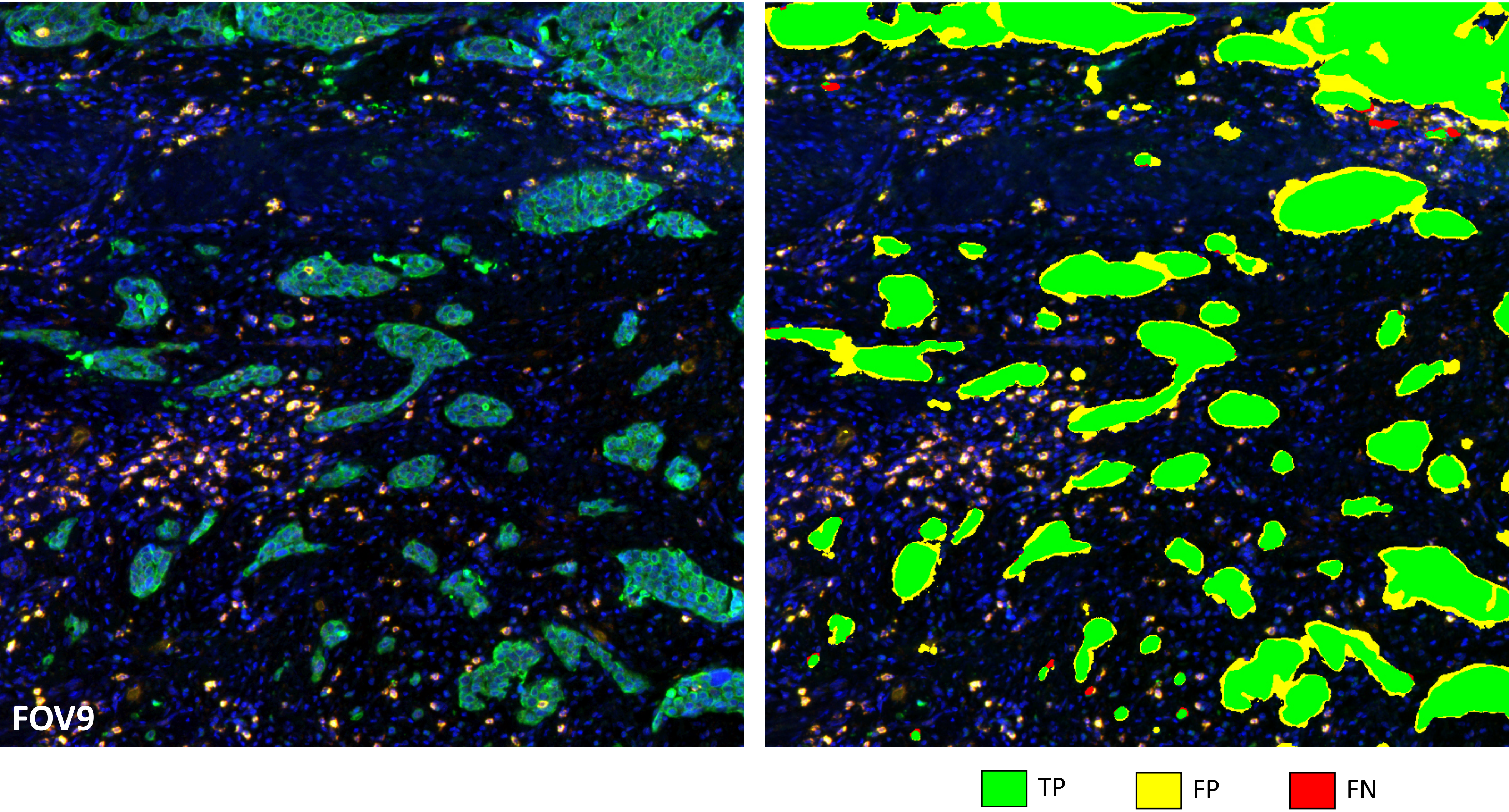
FOV9
TP
FP
FN

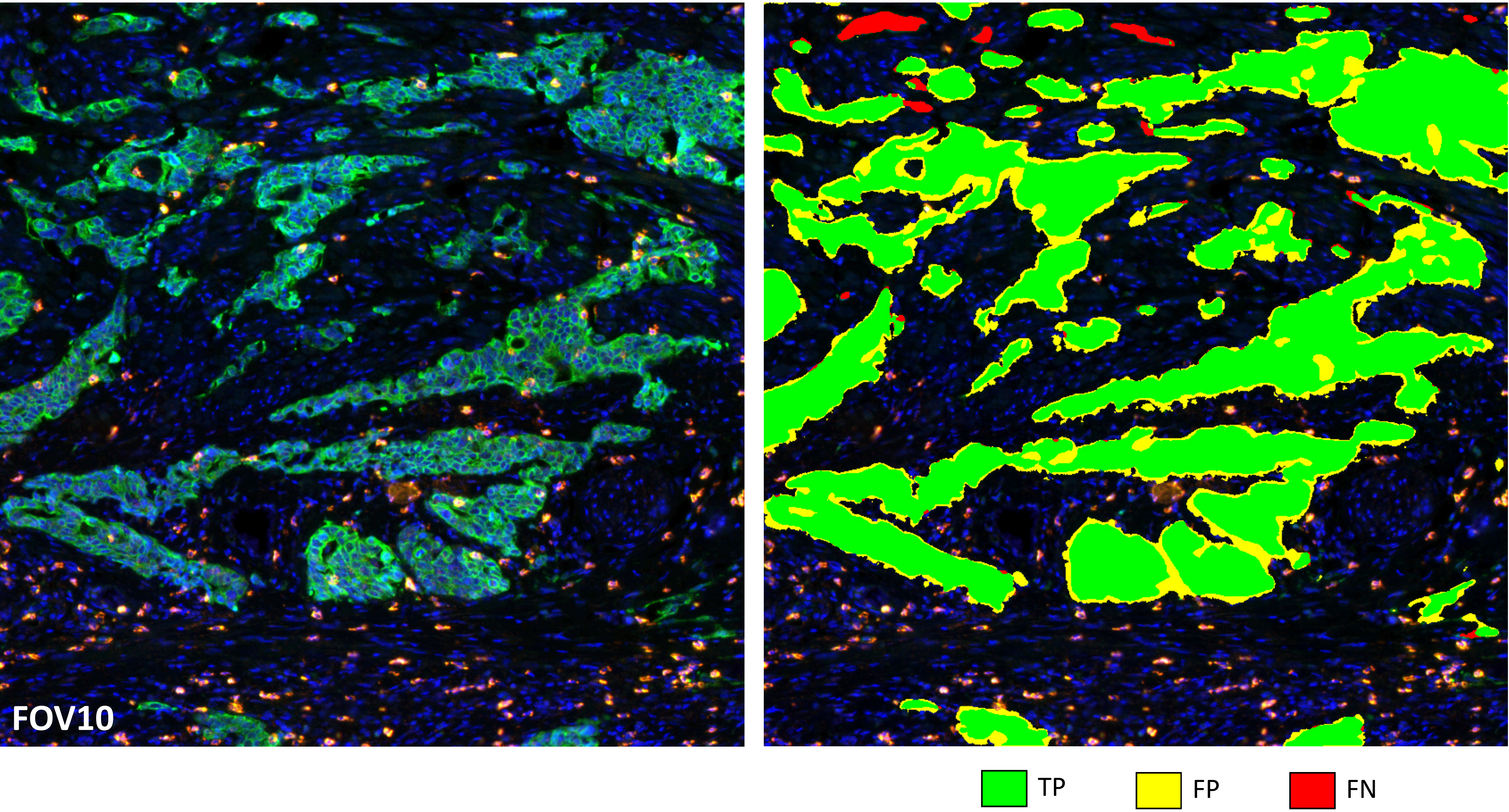
FOV10
TP
FP
FN
