# Supplementary material for: Automated tumour budding quantification by machine learning augments TNM staging in muscle-invasive bladder cancer prognosis
Source: Sci Rep. 2019 Mar 26;9:5174. doi: 10.1038/s41598-019-41595-2 (PMC6435679; doi:10.1038/s41598-019-41595-2)
Supplement: Supplementary file 4 — Supplementary Material M3 [file 41598_2019_41595_MOESM4_ESM.pdf]

Title: Automated tumour budding quantification by machine learning augments TNM staging in muscle-invasive bladder cancer prognosis.

Authors: Brieu Nicolas[1], Gavriel G Christos[2], Nearchou P Ines[2], Harrison J David[2], Schmidt Günter[1] and Caie D Peter[2]*

[1]Definiens AG, Bernhard-Wicki-Straße 5, 80636 München, Germany.

[2]School of Medicine, University of St Andrews, North Haugh, St Andrews, Fife, KY16 9TF, UK.

Brieu Nicolas and Gavriel G Christos contributed equally to this work.

Supplementary Material M3. Qualitative results of the nuclei detection algorithm, on the first 5 FOVs provided in supplementary materials M2-M7. Red dots indicate manually annotated nuclei, yellow dots indicate automatically detected nuclei.

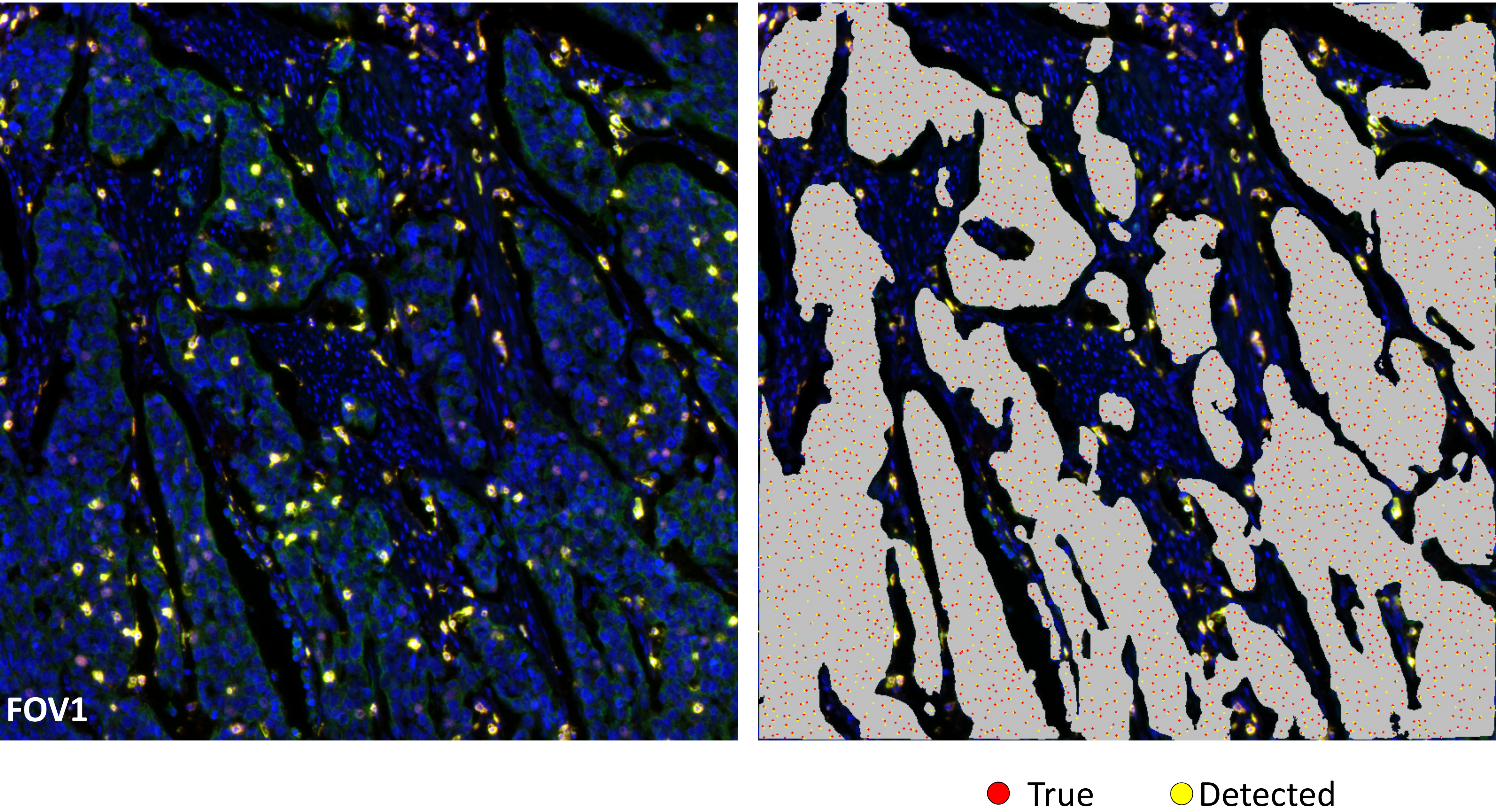
FOV1
True
Detected

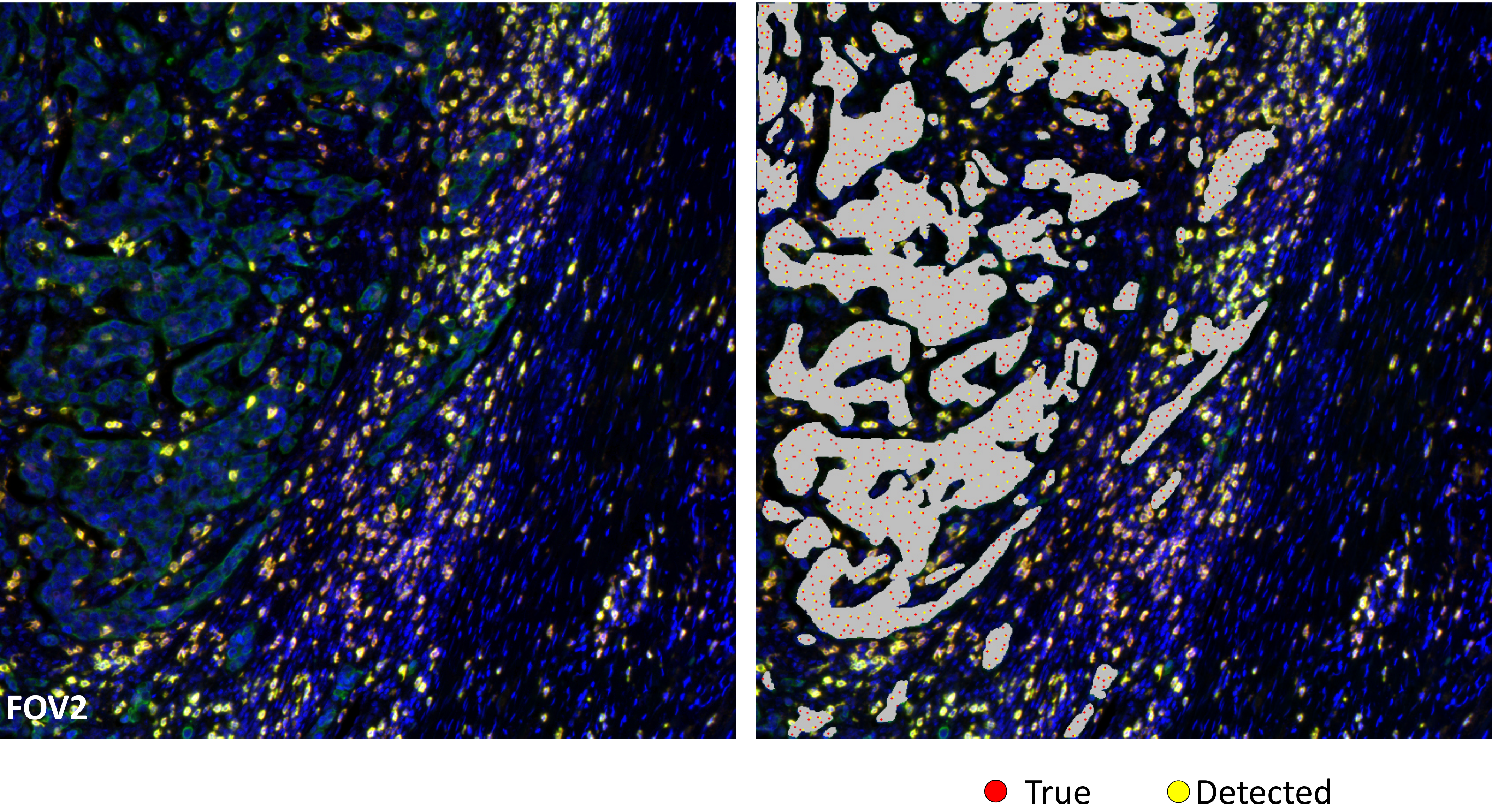
FOV2
True
Detected

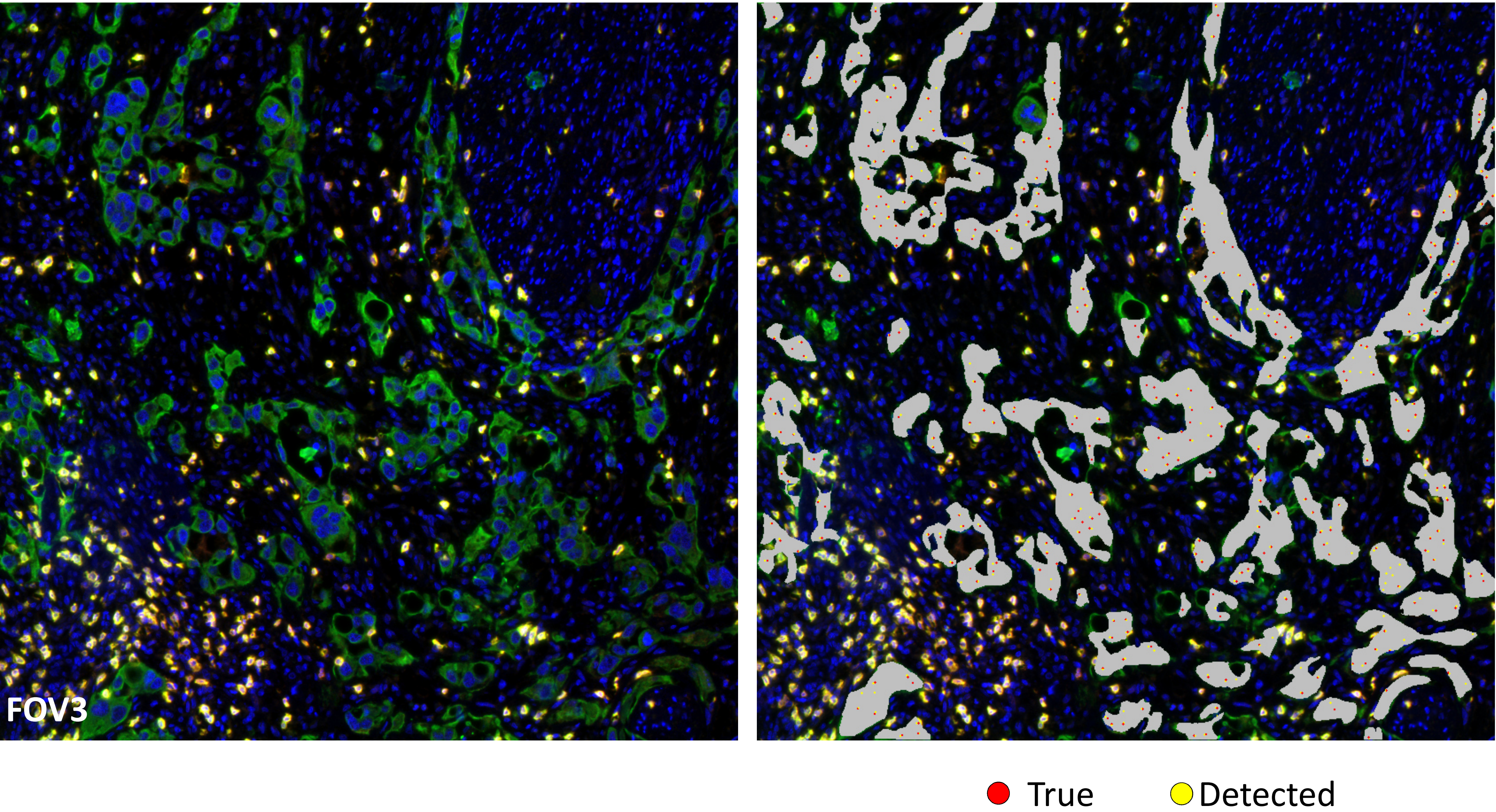

FOV3
True
Detected

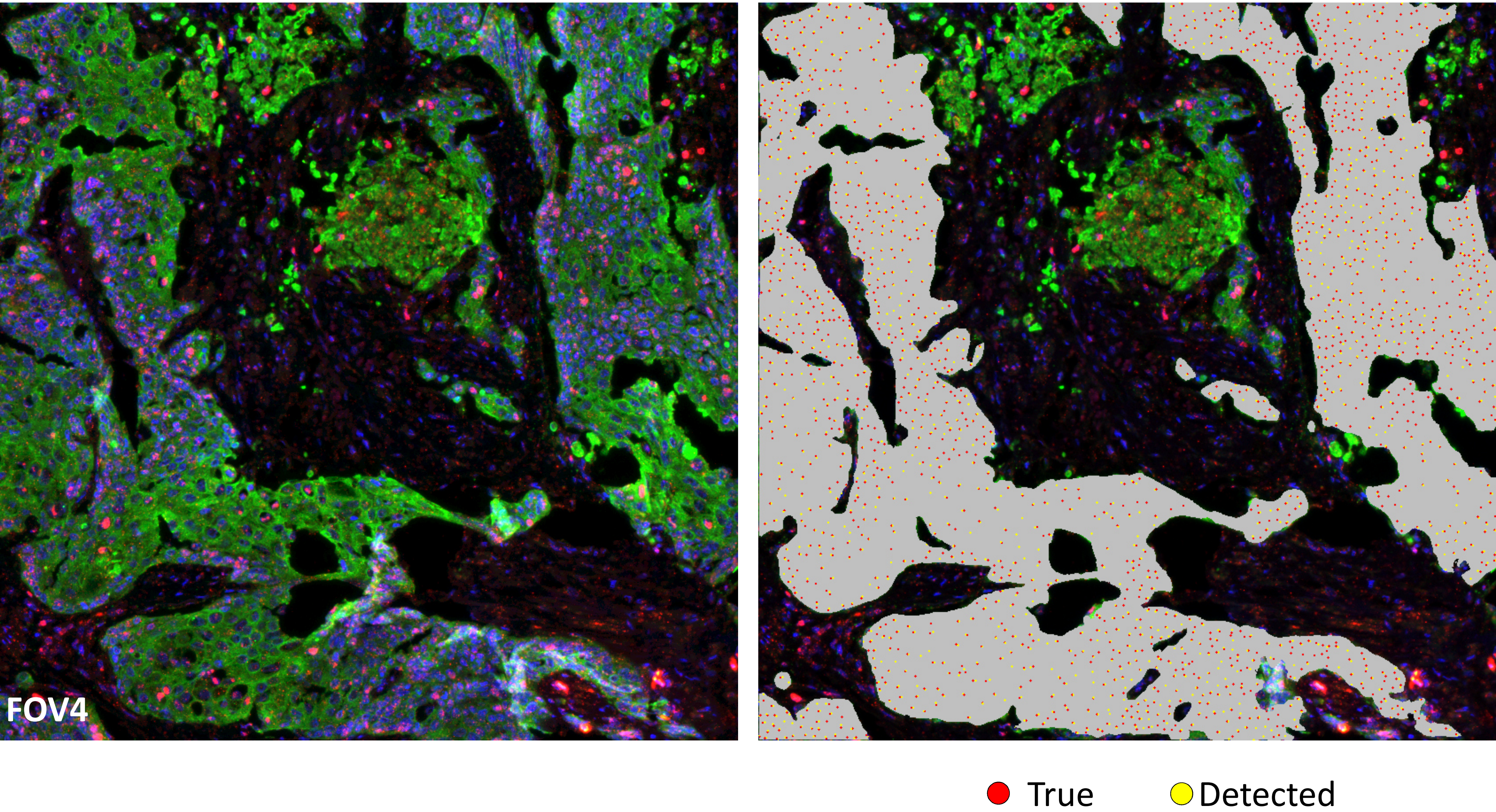
FOV4
True
Detected

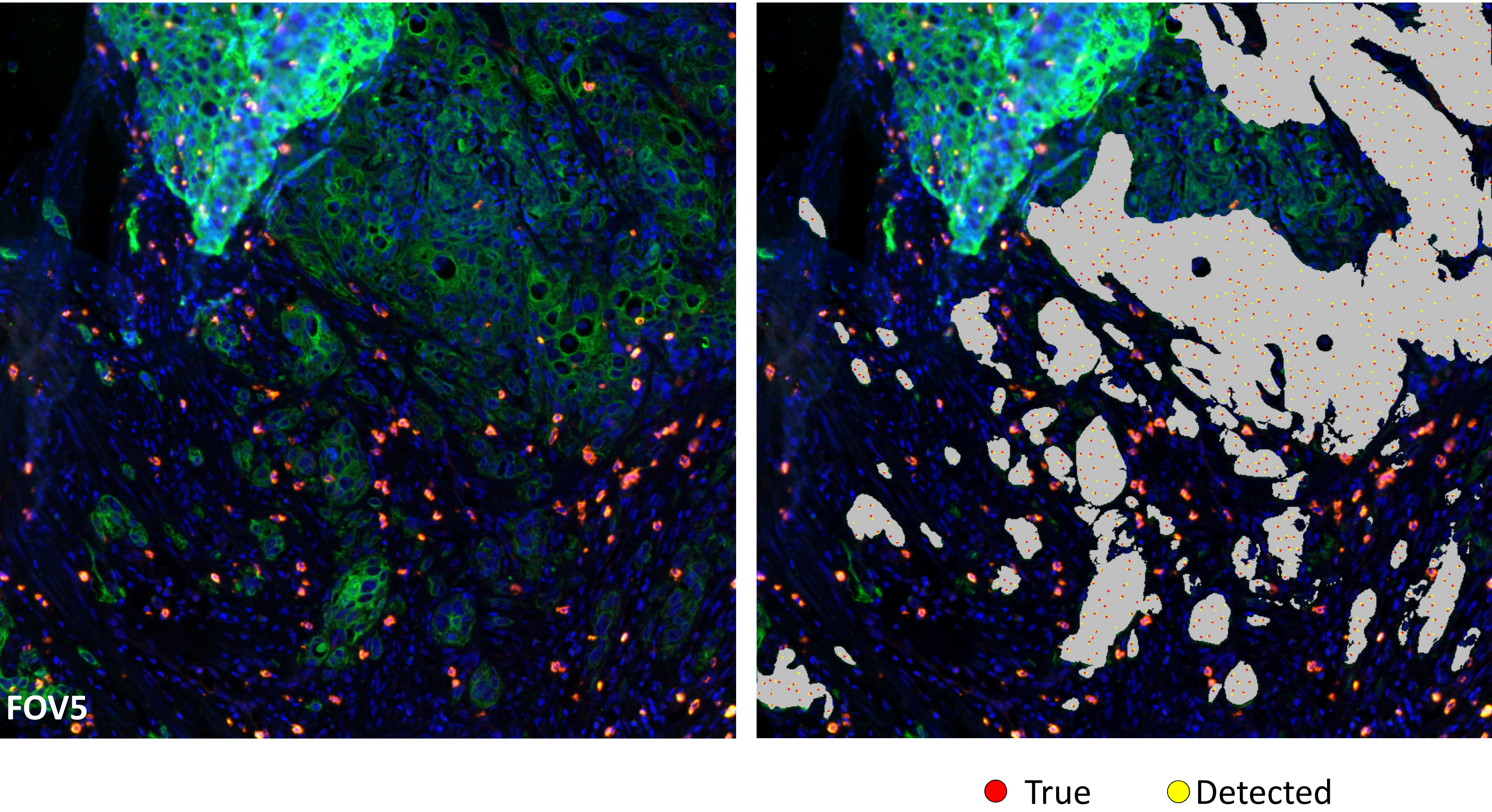
FOV5
True
Detected
