# Supplementary material for: Automated tumour budding quantification by machine learning augments TNM staging in muscle-invasive bladder cancer prognosis
Source: Sci Rep. 2019 Mar 26;9:5174. doi: 10.1038/s41598-019-41595-2 (PMC6435679; doi:10.1038/s41598-019-41595-2)
Supplement: Supplementary file 8 — Supplementary Material M7 [file 41598_2019_41595_MOESM8_ESM.pdf]

Title: Automated tumour budding quantification by machine learning augments TNM staging in muscle-invasive bladder cancer prognosis.

Authors: Brieu Nicolas[1], Gavriel G Christos[2], Nearchou P Ines[2], Harrison J David[2], Schmidt Günter[1] and Caie D Peter[2]*

[1]Definiens AG, Bernhard-Wicki-Straße 5, 80636 München, Germany.

[2]School of Medicine, University of St Andrews, North Haugh, St Andrews, Fife, KY16 9TF, UK.

Brieu Nicolas and Gavriel G Christos contributed equally to this work.

Supplementary Material M7. FOVs used for the quantitative evaluation of the segmentation and detection algorithms. Blue channel is Hoechst, Green channel is panCK and Red channel is an immune cell marker unused in this study. Images were transformed from 16 bits to 8 bits by windowing on the lowest and highest 1% quantiles.

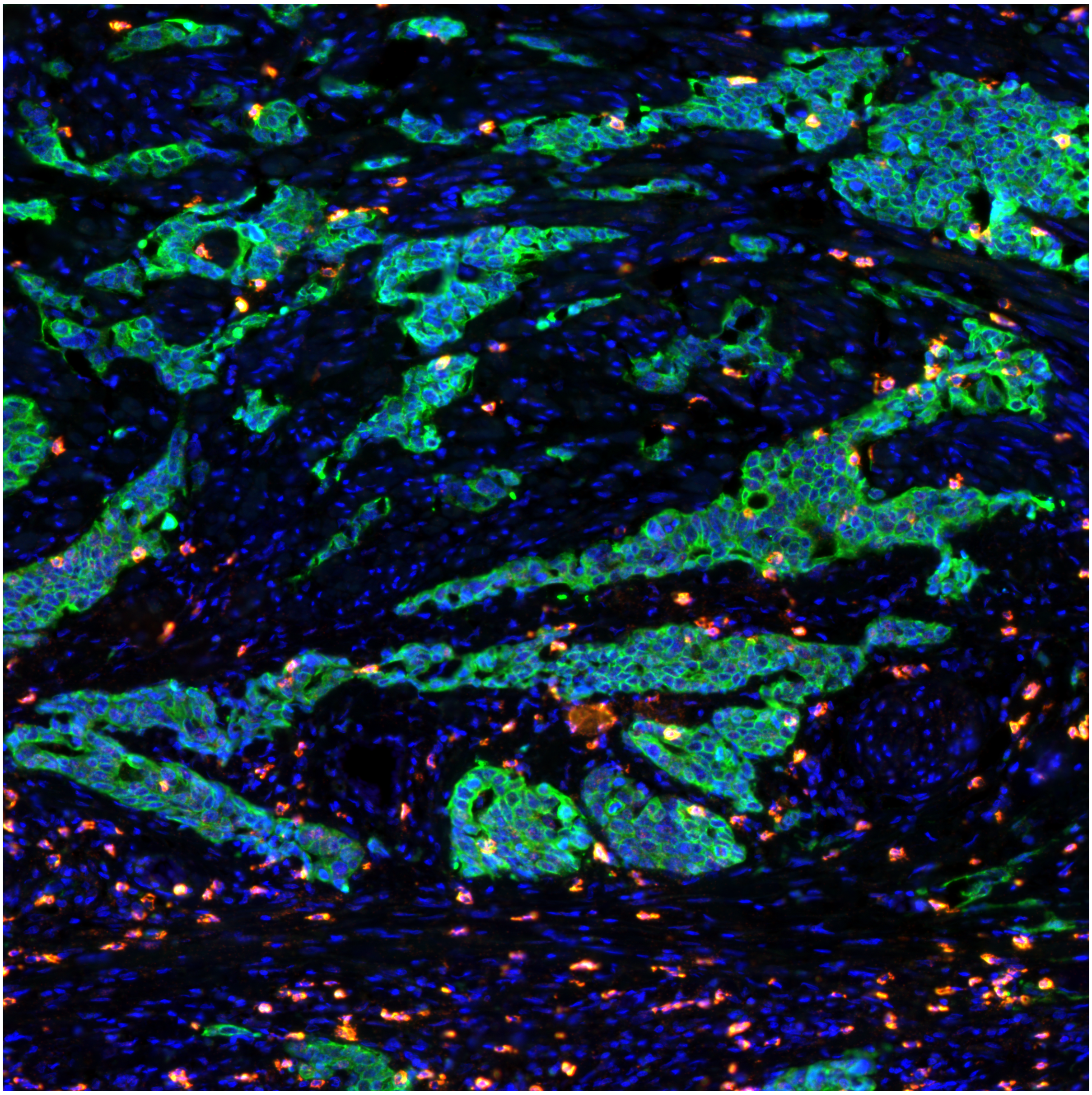

FOV10
